# Supplementary material for: Sequence logic at enhancers governs a dual mechanism of endodermal organ fate induction by FOXA pioneer factors
Source: Nat Commun. 2021 Nov 17;12:6636. doi: 10.1038/s41467-021-26950-0 (PMC8599738; doi:10.1038/s41467-021-26950-0)
Supplement: Supplementary file 1 — Supplementary Information [file 41467_2021_26950_MOESM1_ESM.pdf]

Sequence logic at enhancers governs a dual mechanism of endodermal organ fate induction by FOXA pioneer factors

Geusz, et al.

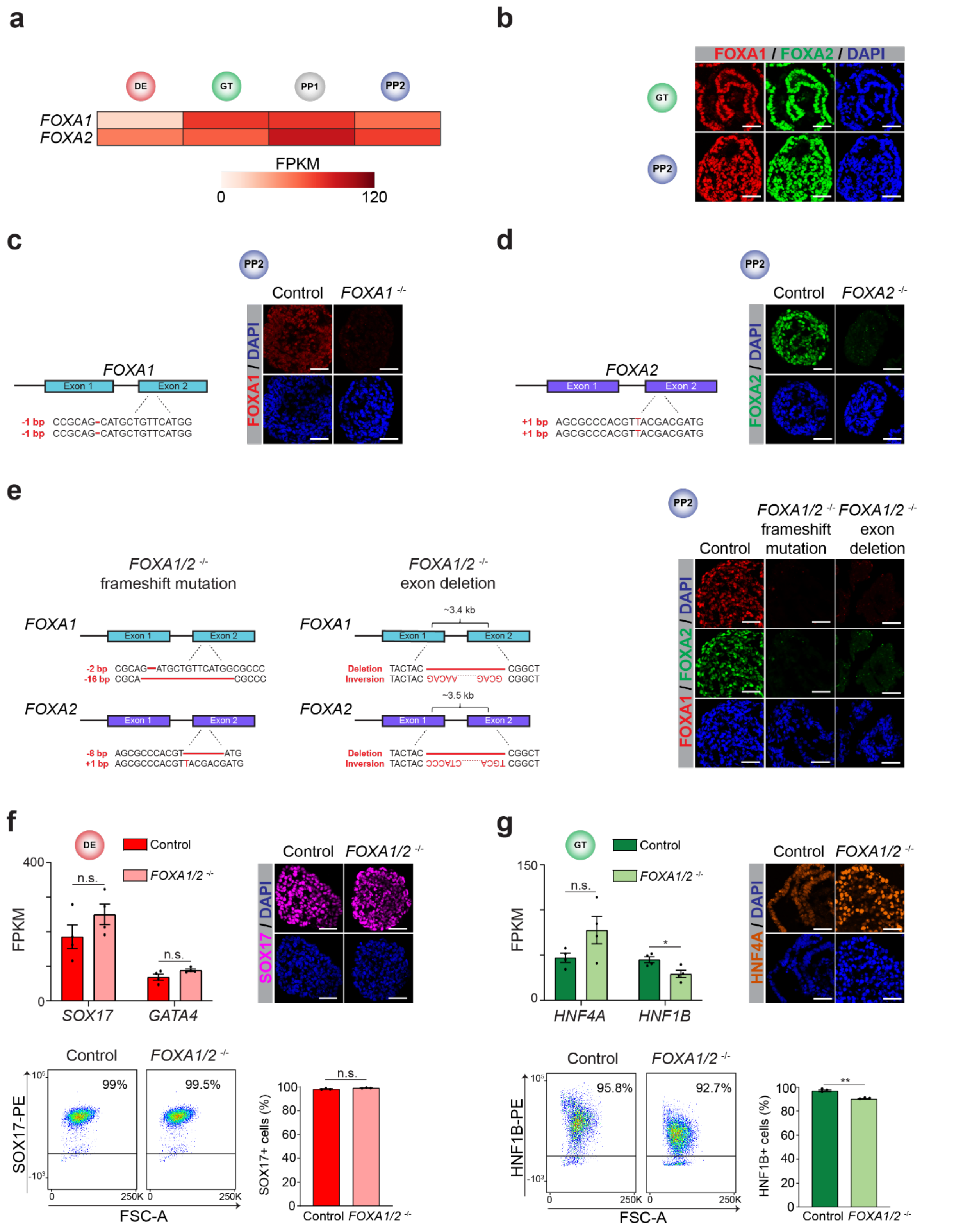

Supplementary Figure 1. FOXA1 and FOXA2 promote pancreatic lineage induction. (**a**) Heatmap showing mRNA expression levels of *FOXA1* and *FOXA2* determined by RNA-seq during pancreatic

differentiation of hESCs. FPKM, Fragments per kilobase per million fragments mapped. (**b**) Immunofluorescent staining of FOXA1 and FOXA2 in GT and PP2. (**c**) Schematic of frameshift mutation in *FOXA1*$^{-/-}$ hESCs (left) and immunofluorescent staining (right) of FOXA1 in PP2. (**d**) Schematic of frameshift mutation in *FOXA2*$^{-/-}$ hESCs (left) and immunofluorescent staining (right) of FOXA2 in PP2. (**e**) Schematic of frameshift mutations and exon deletions in *FOXA1/2*$^{-/-}$ hESCs (left) and immunofluorescent staining (right) of FOXA1 and FOXA2 in PP2. (**f** and **g**) mRNA expression levels determined by RNA-seq (left), immunofluorescent staining (right), and flow cytometry quantification (bottom) in control and *FOXA1/2*$^{-/-}$ DE (**f**) and GT (**g**) cells (n = 4 and n = 3 independent differentiations for RNA-seq and flow cytometry, respectively; qPCR: *P* adj. = 0.116 and 0.104 for *SOX17* and *GATA4*, respectively, in **f** and 0.014 and 0.061 for *HNF1B* and *HNF4A*, respectively, in **g**; DESeq2; flow cytometry: *P* = 0.116 in control compared to *FOXA1/2*$^{-/-}$ DE cells in **f** and 4.5×10^{-3} in control compared to *FOXA1/2*$^{-/-}$ GT cells in **g**; student's t-test, 2 sided). Bar graphs show mean ± S.E.M. FSC-A, forward scatter area. For all immunofluorescence, representative images are shown from n ≥ 2 independent differentiations; scale bars, 50 μm.

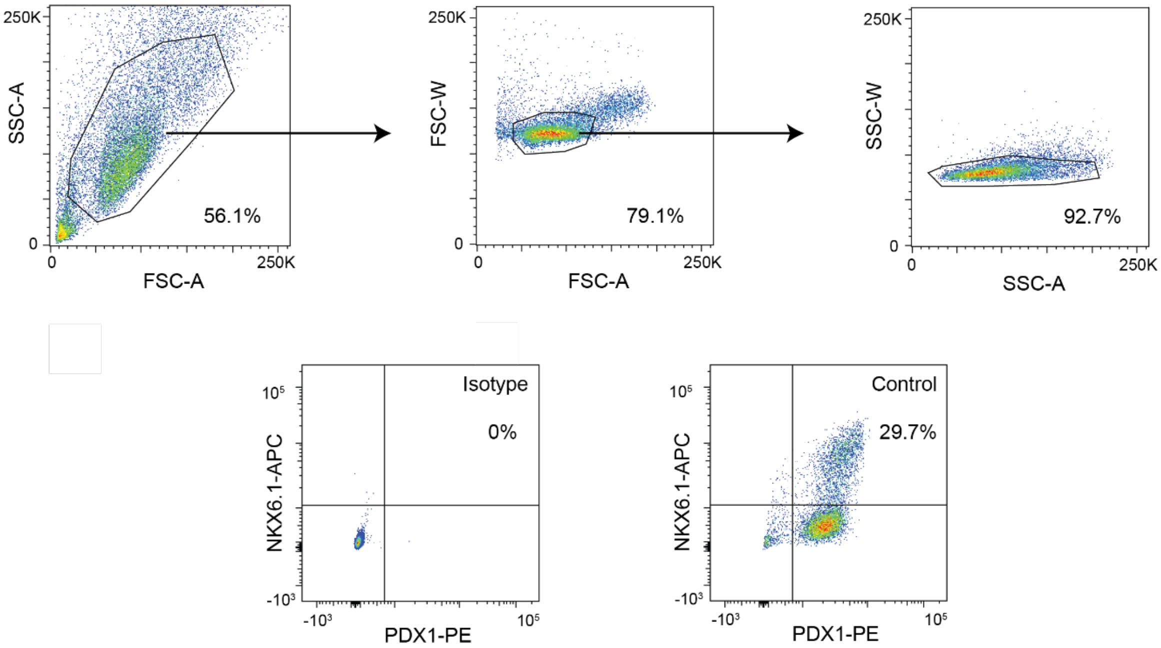

Supplementary Figure 2. Gating strategy for FACS analysis. Cells were gated to identify live cells (FSCA vs SSC-A) and singlets (FSC-A vs FSC-W, SSC-A vs SSC-W). Isotype controls were performed using antibodies against IgG conjugated to corresponding fluorophores.

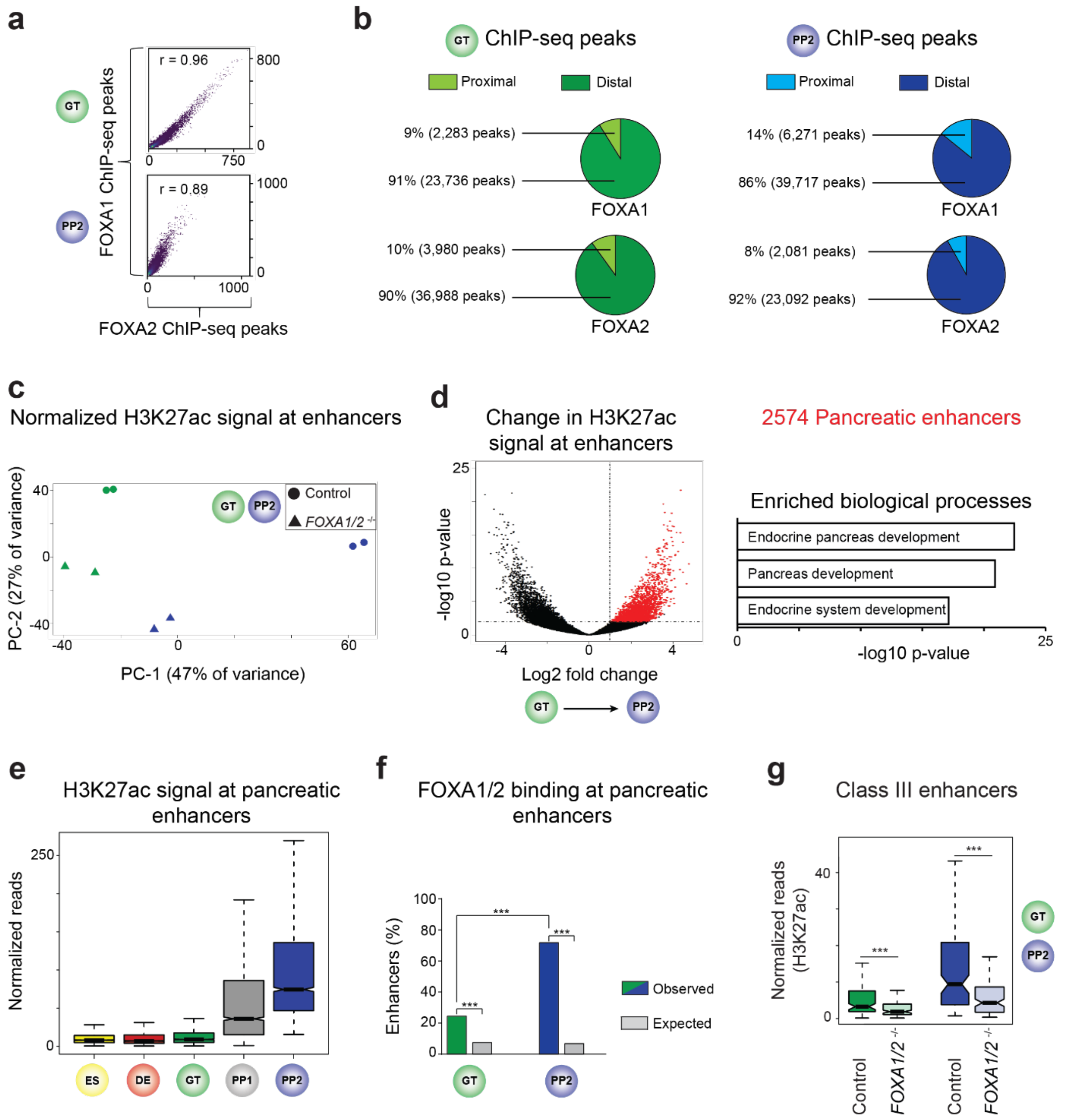

Supplementary Figure 3. FOXA1 and FOXA2 both bind to pancreas-specific enhancers. (**a**) Pearson correlation between FOXA1 and FOXA2 ChIP-seq signal at FOXA1 and FOXA2 peaks in GT and PP2. (**b**) Percentage of FOXA1 and FOXA2 peaks located proximal (≤ 2.5 kb) or distal (> 2.5 kb) to nearest annotated TSS. (**c**) Principal component analysis showing variance in distal (> 2.5 kb from TSS) H3K27ac signal between control and *FOXA1/2*$^{-/-}$ cells in GT and PP2. Each point represents one biological replicate. (**d**) Volcano plot showing identification of pancreatic enhancers based on increase in H3K27ac signal from GT to PP2 (≥ 2-fold increase, *P* adj. < 0.05 at sites > 2.5 kb from TSS; DESeq2). Enriched gene ontology terms of genes linked to pancreatic enhancers using GREAT. (**e**) Box plots of H3K27ac ChIP-seq counts at pancreatic enhancers. (**f**) Enrichment of pancreatic enhancers for FOXA1 or FOXA2 peaks compared to random genomic regions at GT and PP2 ($P < .0001$ and $P < .0001$, respectively; permutation test). Pancreatic enhancers are enriched for FOXA1/2 peaks at PP2 compared to GT ($P < 2.2 \times 10^{-16}$, Fisher's exact test, 2-sided). (**g**) Box plots of H3K27ac ChIP-seq counts at class III pancreatic enhancers in control and *FOXA1/2*$^{-/-}$ GT and PP2 cells ($P = 1.47 \times 10^{-5}$ and 3.17×10^{-6} for control versus *FOXA1/2*$^{-/-}$ at class III

enhancers in GT and PP2, respectively; Wilcoxon rank sum test, 2-sided). All box plots are centered on median, with box encompassing 25th-75th percentile and whiskers extending up to 1.5 interquartile range. All ChIP-seq experiments, n = 2 replicates from independent differentiations.

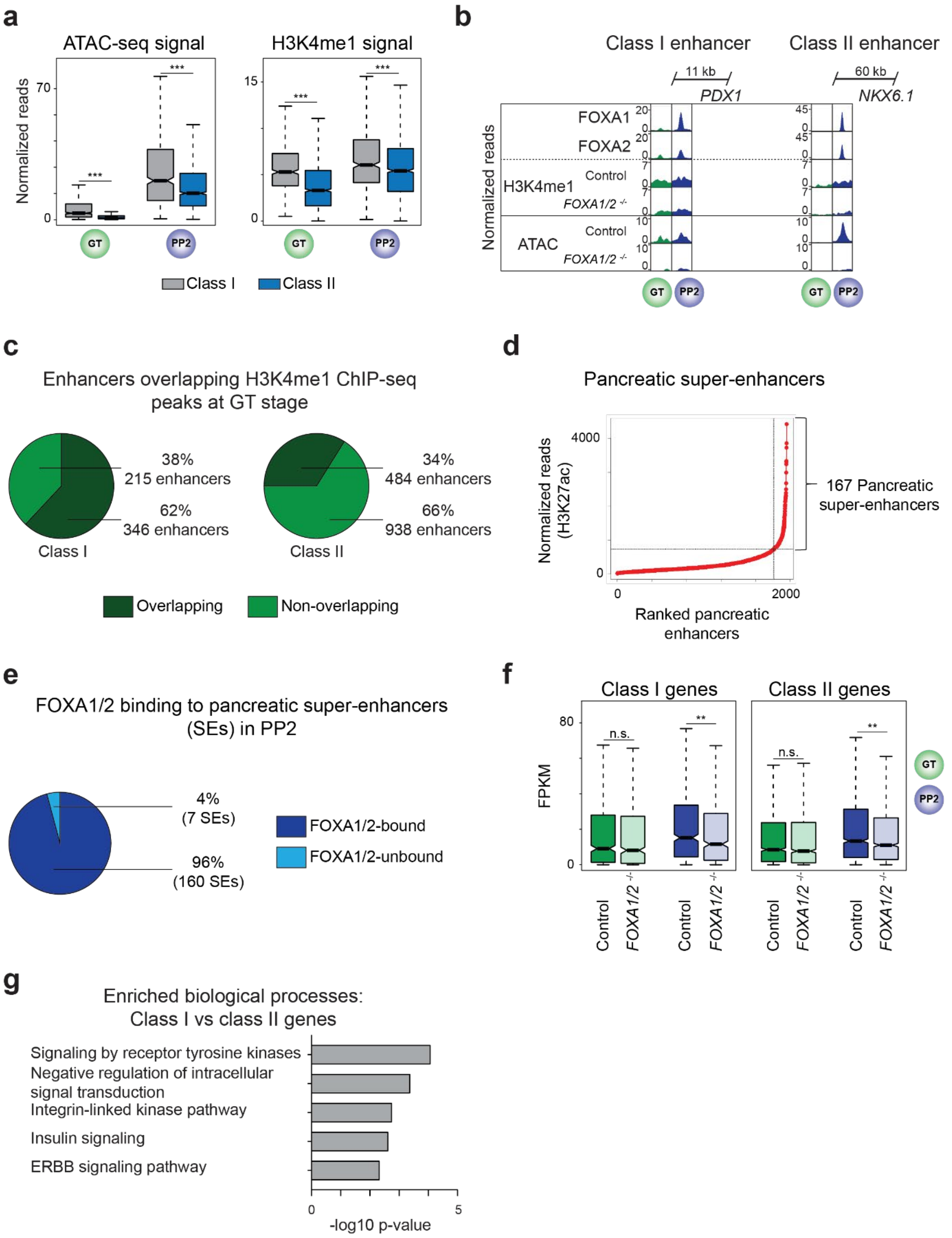

Supplementary Figure 4. Characterization of class I and class II pancreatic enhancers. (**a**) Box plots of ATAC-seq and H3K4me1 ChIP-seq counts at class I and class II enhancers in GT and PP2 (*P* = < 2.2 x

10^{-16}, 1.11 x 10^{-14}, < 2.2 x 10^{-16}, and 1.07 x 10^{-7} for comparisons of ATAC-seq signal in GT and PP2 and comparisons of H3K4me1 ChIP-seq signal in GT and PP2, respectively; Wilcoxon rank sum test, 2-sided). (**b**) Genome browser snapshots showing FOXA1, FOXA2, H3K4me1 ChIP-seq, and ATAC-seq signal at class I enhancer near *PDX1* and class II enhancer near *NKX6.1* in GT and PP2 cells. Approximate distance between enhancer and gene body is indicated. (**c**) Percentage of class I and class II enhancers overlapping H3K4me1 ChIP-seq peaks (within 1 kb from peak) in GT. (**d**) Identification of pancreatic super-enhancers by ranking 2574 pancreatic enhancers based on H3K27ac ChIP-seq signal in PP2. (**e**) Percentage of pancreatic super-enhancers containing FOXA1 and/or FOXA2 ChIP-seq peaks in PP2. (**f**) Box plots of mRNA levels (FPKM, fragments per kilobase per million fragments mapped) in control and *FOXA1/2*$^{-/-}$ GT and PP2 cells for genes linked to pancreatic class I and class II enhancers (n = 4 independent differentiations; P = 0.182, 4.82 x 10^{-3}, 0.067, and 1.21 x 10^{-3} for control versus *FOXA1/2*$^{-/-}$ of class I genes in GT, class I genes in PP2, class II genes in GT, and class II genes in PP2, respectively; Wilcoxon rank sum test, 2-sided). (**g**) Gene ontology terms enriched in genes associated with class I compared to class II enhancers. All box plots are centered on median, with box encompassing 25th-75th percentile and whiskers extending up to 1.5 interquartile range. All ChIP-seq and ATAC-seq experiments, n = 2 replicates from independent differentiations.

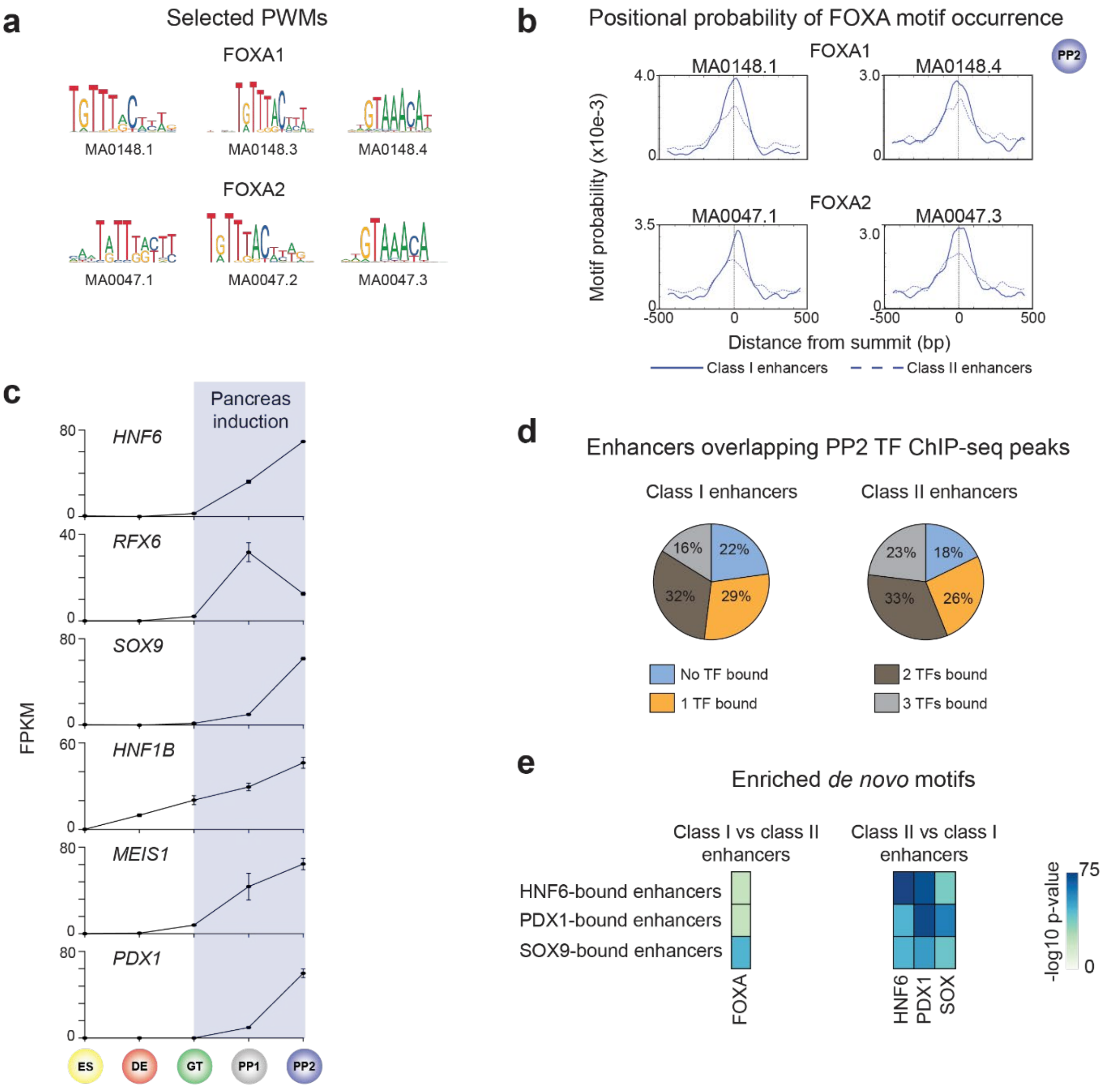

Supplementary Figure 5. Class I and class II pancreatic enhancers exhibit distinct enhancer architecture. (**a**) Selected FOXA1 and FOXA2 motifs and associated position weight matrices (PWMs) obtained from JASPAR. (**b**) Probability (occurrence per base pair) of FOXA1 (MA0148.1 and MA0148.4) and FOXA2 (MA0047.1 and MA0047.3) motifs relative to ATAC-seq peak summits at class I (solid line) and class II (dashed line) enhancers. ATAC-seq peak summits at class I enhancers are enriched for occurrences compared to summits at class II enhancers ($P = 8.4 \times 10^{-15}$, 1.6×10^{-3}, 1.3×10^{-3}, and 2.1×10^{-5} for MA0148.1, MA0148.4, MA0047.1, and MA0047.3, respectively; Fisher's exact test, 1-sided). (**c**) mRNA expression levels of pancreatic transcription factors (TF) determined by RNA-seq. Data are shown as mean fragments per kilobase per million fragments mapped (FPKM) ± S.E.M. in ES, DE, GT, PP1, and PP2 (n = 3 independent differentiations). (**d**) Percentage of class I and class II enhancers overlapping HNF6, PDX1, and SOX9 ChIP-seq peaks (within 100 bp from peak) in PP2. (**e**) Heatmap showing enriched *de novo* TF binding motifs at HNF6-, PDX1-, and SOX9-bound class I against a background of HNF6-, PDX1-, and SOX9-bound class II enhancers and vice versa. Fisher's exact test, 1-sided, corrected for multiple comparisons.

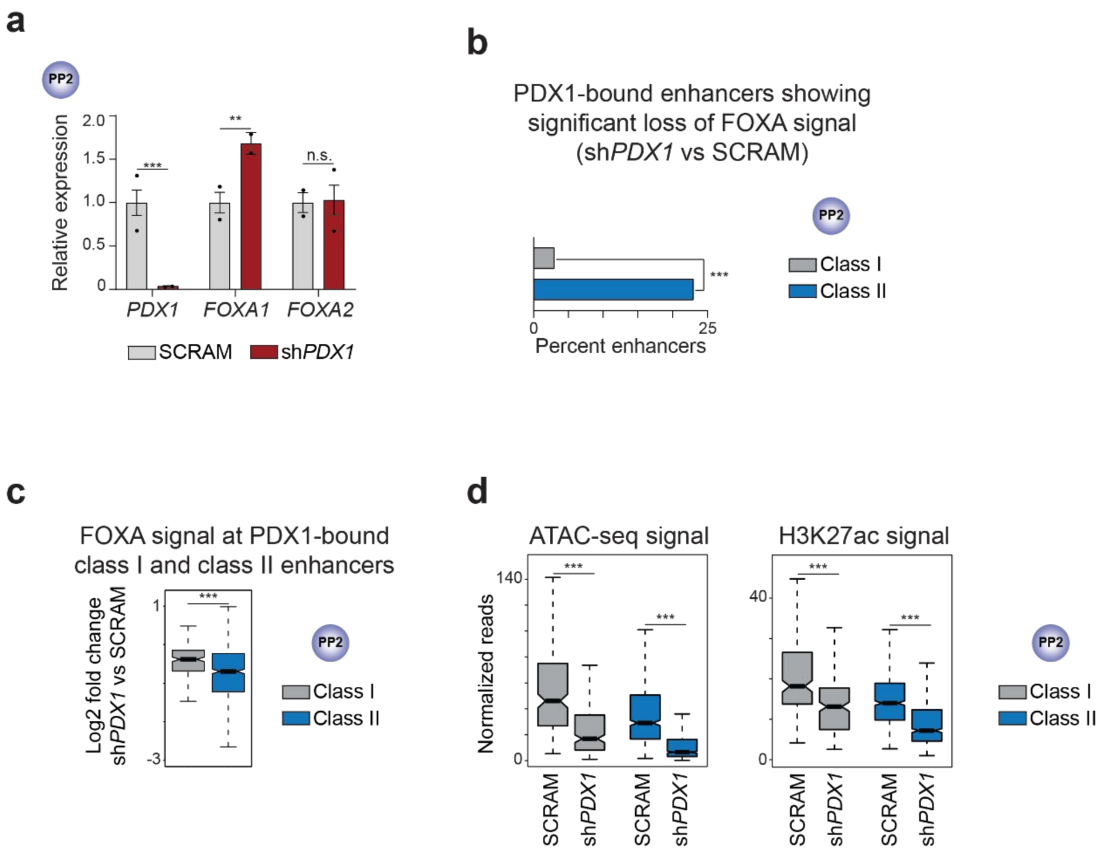

Supplementary Figure 6. Characterization of class I and class II enhancers in PDX1-deficient pancreatic progenitors. (**a**) qPCR analysis of *PDX1*, *FOXA1*, and *FOXA2* in PP2 cells differentiated from hESCs transduced with a scrambled control (SCRAM) or *PDX1* shRNA (sh*PDX1*) lentivirus (n = 2 independent differentiations; P = 5.90 x 10^{-5}, 2.74 x 10^{-3}, and 0.883 for *PDX1*, *FOXA1*, and *FOXA2*, respectively, in SCRAM compared to sh*PDX1* PP2 cells; student's t-test, 2-sided). Bar graph shows mean ± S.E.M. (**b**) Percentage of PDX1-bound class I and class II enhancers exhibiting a significant loss of FOXA1/2 ChIP-seq signal (≥ 2-fold decrease, P adj. < 0.05) in sh*PDX1* vs SCRAM PP2 (P < 2.2 x 10^{-16}; Fisher's exact test, 2-sided). (**c**) Box plot of fold change in FOXA1/2 ChIP-seq signal at PDX1-bound class I and class II enhancers in sh*PDX1* vs SCRAM PP2 cells. (P = < 2.2 x 10^{-16}; Wilcoxon rank sum test, 2-sided). (**d**) Box plots of ATAC-seq and H3K27ac ChIP-seq counts at class I and class II pancreatic enhancers in SCRAM or sh*PDX1* PP2 cells (P = < 2.2 x 10^{-16}, < 2.2 x 10^{-16}, 7.03 x 10^{-13}, and < 2.2 x 10^{-16} for SCRAM versus sh*PDX1* of ATAC signal at class I enhancers, ATAC signal at class II enhancers, H3K27ac signal at class I enhancers, and H3K27ac signal at class II enhancers, respectively; Wilcoxon rank sum test, 2-sided). All box plots are centered on median, with box encompassing 25th-75th percentile and whiskers extending up to 1.5 interquartile range. All ChIP-seq and ATAC-seq experiments, n = 2 replicates from independent differentiations.

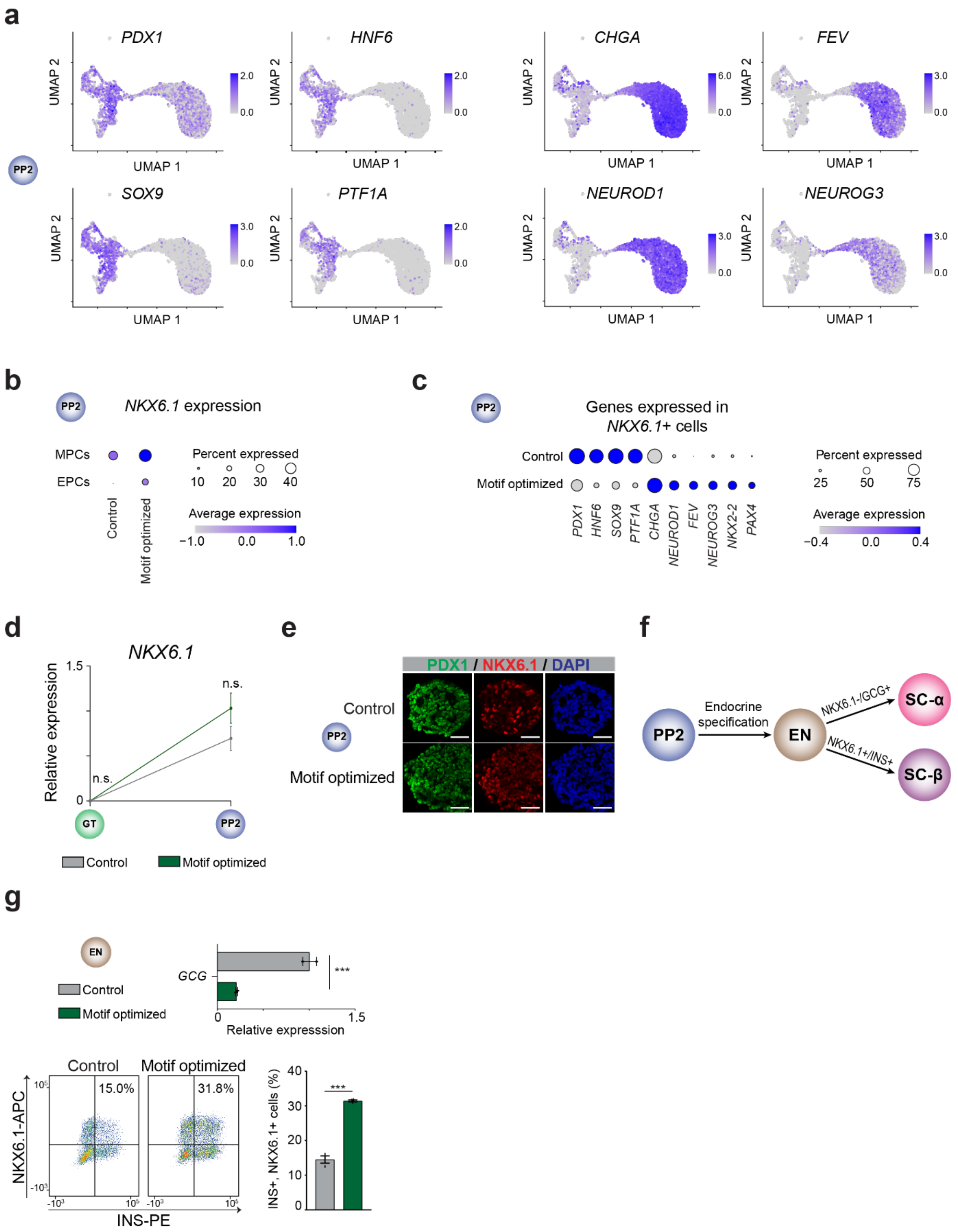

Supplementary Figure 7. Optimization of FOXA binding motifs alters target gene expression and cell fate. (**a**) Feature plots of single cell RNA-seq data showing expression of selected marker genes across cell populations at PP2. UMAPs shown are integrated across control and motif optimized cells. (**b**) Dot plot

showing expression levels of *NKX6.1* across cell populations in control and motif optimized PP2 cells. (**c**) Dot plot showing expression levels of select marker genes in *NKX6.1*-expressing cells in control and motif optimized PP2 cells. (**d**) qPCR analysis of *NKX6.1* in control and motif optimized GT and PP2 cells (n = 3 independent differentiations; *P* = 0.39 and 0.19 for comparisons at GT and PP2, respectively; student's t-test, 2-sided). Line graph shows mean ± S.E.M. (**e**) Immunofluorescent staining for PDX1 and NKX6.1 in PP2 control and motif optimized cells. Scale bars, 50 µm. Representative images are shown from n ≥ 2 independent differentiations. (**f**) Schematic illustrating differentiation from the late pancreatic progenitor stage (PP2) to the endocrine progenitor stage (EN). (**g**) qPCR analysis of *GCG* (top), and flow cytometry quantification (bottom) of insulin$^+$ and NKX6.1$^+$ cells in control and motif optimized EN cells (qPCR: n = 2 independent differentiations, each plotted point represents the average of 3 technical replicates, *P* = 8.9 x 10^{-3}; flow cytometry: n = 3 independent differentiations, *P* = 2.2 x 10^{-3}; student's t-test, 2-sided). Bar graphs show mean ± S.E.M. Multipotent pancreatic progenitor cells, MPCs; early endocrine progenitor cells, EPCs.

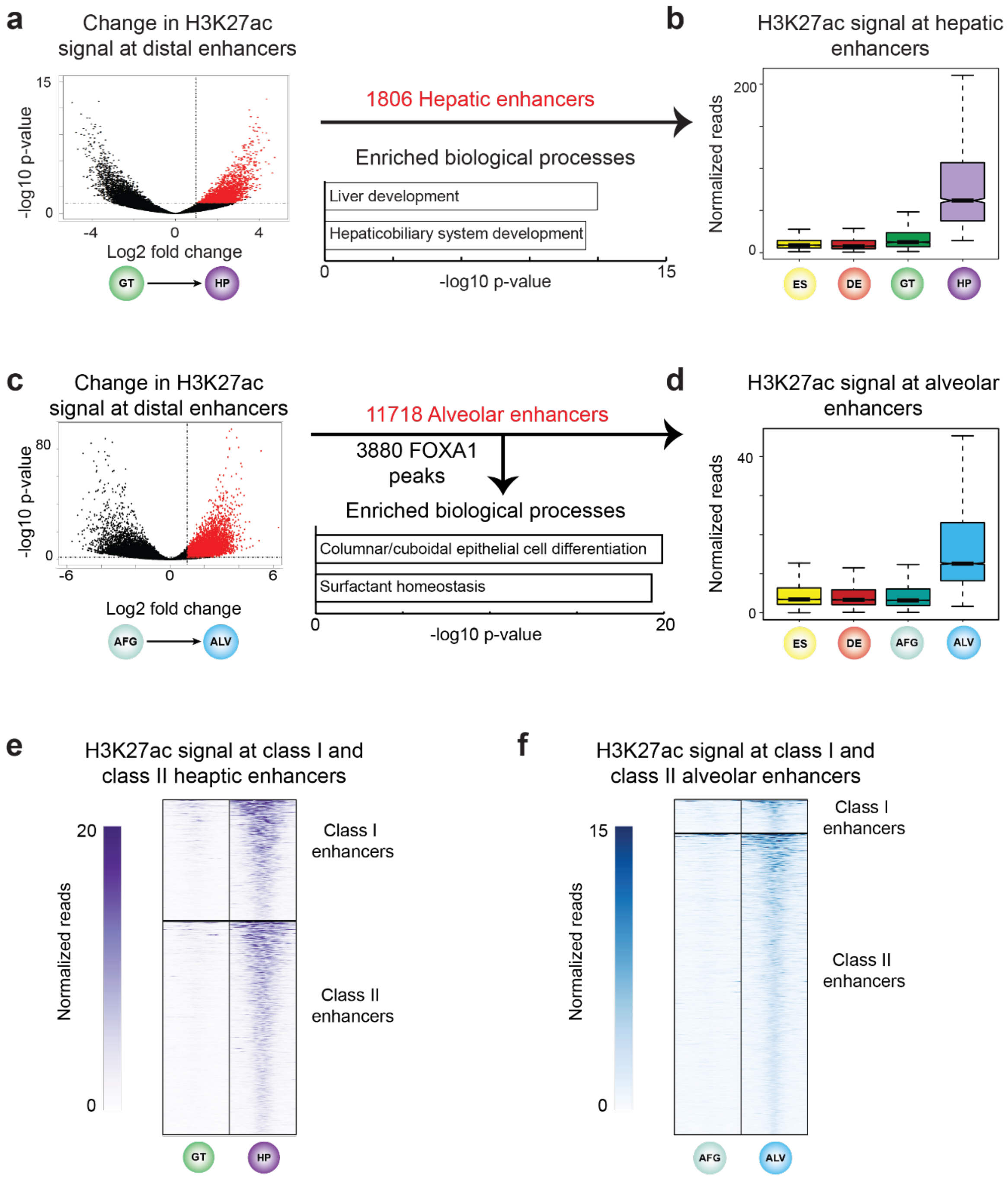

Supplementary Figure 8. Identification and characterization of hepatic and alveolar enhancers. (**a**) Volcano plot showing identification of hepatic enhancers based on increase in H3K27ac signal from GT to HP (≥ 2-fold increase, *P* adj. < 0.05 at sites > 2.5 kb from TSS; DESeq2). Enriched gene ontology terms of genes linked to hepatic enhancers using GREAT. (**b**) Box plots of H3K27ac ChIP-seq counts at hepatic enhancers. (**c**) Volcano plot showing identification of alveolar enhancers based on increase in H3K27ac signal from AFG to ALV (≥ 2-fold increase, *P* adj. < 0.05 at sites > 2.5 kb from TSS; DESeq2). Enriched gene ontology terms of genes linked to alveolar enhancers using GREAT. (**d**) Box plots of H3K27ac ChIP-seq counts at alveolar enhancers. (**e** and **f**) Heatmaps showing density of H3K27ac ChIP-seq reads at hepatic (**e**) and alveolar (**f**) class I and class II enhancers in GT and HP (**e**) and AFG and ALV (**f**). Heatmaps

are centered on H3K27ac peaks and span 5 kb. All box plots are centered on median, with box encompassing 25th-75th percentile and whiskers extending up to 1.5 interquartile range. All ChIP-seq experiments, n = 2 replicates from independent differentiations.

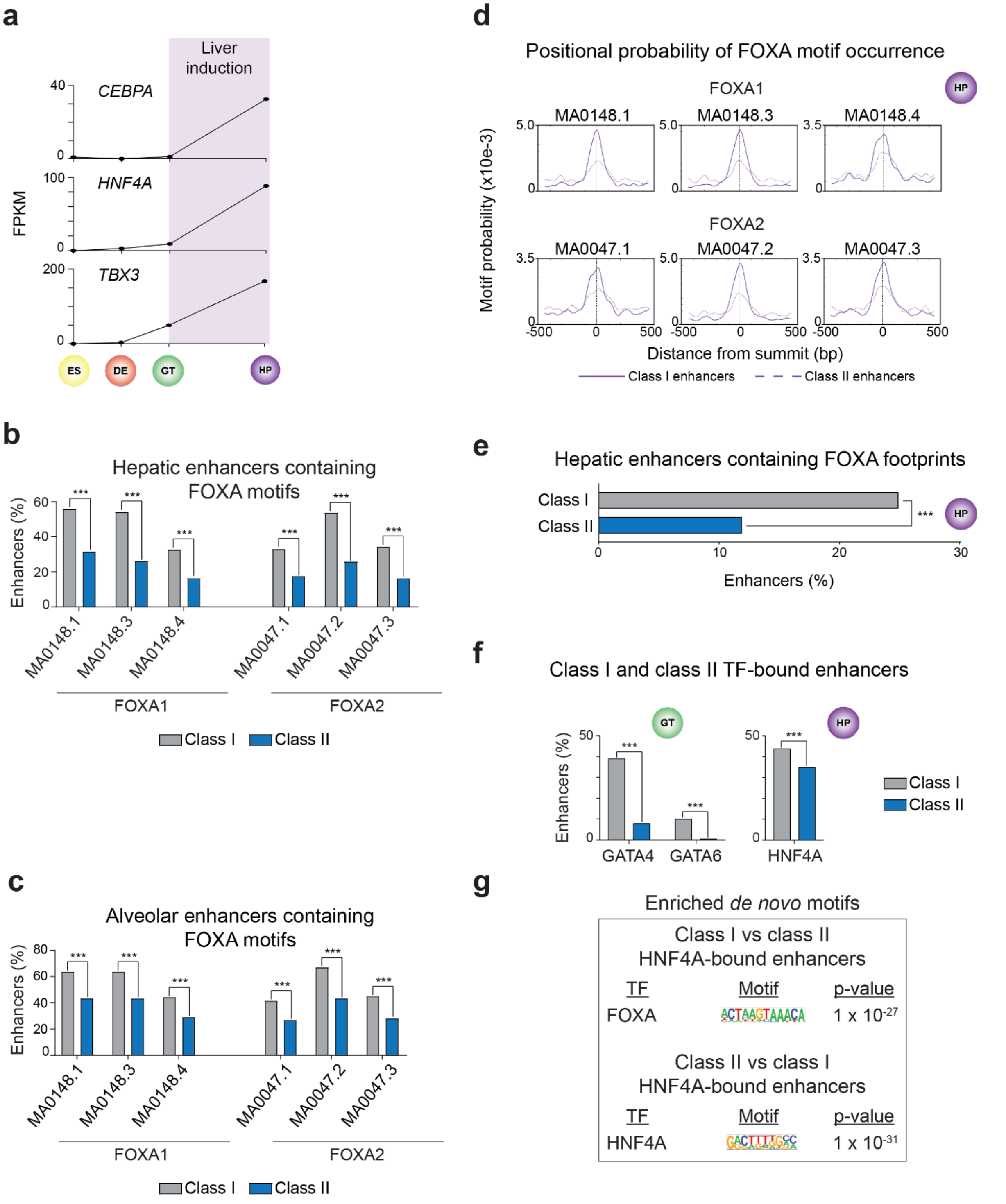

Supplementary Figure 9. FOXA1/2 binding sites at class I and class II hepatic and alveolar enhancers differ in DNA sequence. (**a**) mRNA expression levels of hepatic transcription factors (TF) determined by RNA-seq. Data are shown as mean fragments per kilobase per million fragments mapped (FPKM) ± S.E.M. in ES, DE, GT (n = 3 independent differentiations), and HP (n = 1 differentiation). (**b** and **c**) Percentage of class I and class II hepatic (**b**) and alveolar (**c**) enhancers with at least one occurrence of selected FOXA1 and FOXA2 motifs (*P* = < 2.2×10^{-16}, < 2.2×10^{-16}, 2.42×10^{-12}, 7.81×10^{-11}, < 2.2×10^{-16},

and 1.32 x 10^{-14} for comparisons of occurrences of MA0148.1, MA0148.3, MA0148.4, MA0047.1, MA0047.2, and MA0047.3, respectively, at hepatic enhancers. P = 4.88 x 10^{-13}, 2.11 x 10^{-13}, 6.17 x 10^{-9}, 8.89 x 10^{-9}, 2.2 x 10^{-16}, and 9.75 x 10^{-11} for comparisons of occurrences of MA0148.1, MA0148.3, MA0148.4, MA0047.1, MA0047.2, and MA0047.3, respectively, at alveolar enhancers. Fisher's exact test, 2-sided). (**d**) Probability (occurrences per base pair) of FOXA1 (MA0148.1, MA0148.3, MA0148.4) and FOXA2 (MA0047.1, MA0047.2, MA0047.3) motifs relative to ATAC-seq peak summits at class I (solid line) and class II (dashed line) hepatic enhancers. ATAC-seq peak summits at class I enhancers are enriched for occurrences compared to summits at class II enhancers (P = 4.3 x 10^{-14}, 3.3 x 10^{-19}, 2.0 x 10^{-3}, 5.5 x 10^{-4}, 6.7 x 10^{-17}, and 2.1 x 10^{-5} for MA0148.1, MA0148.3, MA0148.4, MA0047.1, MA0047.2, and MA0047.3, respectively; Fisher's exact test, 1-sided). (**e**) Percentage of hepatic class I and class II enhancers containing FOXA TF ATAC-seq footprints in HP (P = 1.01 x 10^{-10} for comparison of class I and class II enhancers; Fisher's exact test, 2-sided). (**f**) Percentage of hepatic class I and class II enhancers overlapping GATA4 and GATA6 ChIP-seq peaks in GT and HNF4A ChIP-seq peaks (within 100 bp from peak) in HP (P = < 2.2 x 10^{-16}, < 2.2 x 10^{-16}, and 2.51 x 10^{-4} for comparisons of GATA4, GATA6, and HNF4A, respectively; Fisher's exact test). (**g**) Enriched *de novo* TF binding motifs at HNF4A-bound class I against a background of HNF4A-bound class II enhancers and vice versa. Fisher's exact test, 1-sided, corrected for multiple comparisons. All ChIP-seq experiments, n = 2 replicates from independent differentiations.

Supplementary Tables

Supplementary Table 1. Reagents used for maintenance and differentiation of Cyt49 hESCs.

Reagent	Manufacturer	Catalog Number
2-Mercaptoethanol	Thermo Fisher Scientific	Cat# 21985023
Accutase	Thermo Fisher Scientific	Cat# 00-4555-56
Activin A	R&D Systems	Cat# 338-AC/CF
B-27 supplement	Thermo Fisher Scientific	Cat# 17504044
DMEM High Glucose	VWR	Cat# 16750-082
DMEM/F12 [-] L-glutamine	VWR	Cat# 15-090-CV
Fetal Bovine Serum	Thermo Fisher Scientific	Cat# MT35011CV
GlutaMAX	Thermo Fisher Scientific	Cat# 35050061
Human AB Serum	Valley Biomedical	Cat# HP1022
Insulin-Transferrin-Selenium (ITS)	Thermo Fisher Scientific	Cat# 41400045
KAAD-Cyclopamine	Toronto Research Chemicals	Cat# K171000
KnockOut SR XenoFree	Thermo Fisher Scientific	Cat# A1099202
Matrigel®	Corning	Cat# 356231
Non-Essential Amino Acids	Thermo Fisher Scientific	Cat# 11140050
Penicillin-Streptomycin	Thermo Fisher Scientific	Cat# 15140122
Recombinant EGF	R&D Systems	Cat# 236-EG
Recombinant Heregulinβ-1	Peprotech	Cat# 100-03
Recombinant human BMP4	R&D Systems	Cat#314-BP
Recombinant KGF/FGF7	R&D Systems	Cat# 251-KG
Recombinant Mouse Wnt3A	R&D Systems	Cat# 1324-WN/CF
Recombinant Noggin	R&D Systems	Cat# 3344NG
RPMI 1640 [-] L-glutamine	VWR	Cat# 15-040-CV
TGF-β RI Kinase Inhibitor IV	Calbiochem	Cat# 616454
TTNPB	Enzo Life Sciences	Cat# BML-GR105

Supplementary Table 2. Reagents used for maintenance and differentiation of H1 hESCs.

Reagent	Manufacturer	Catalog Number
3,3',5-Triiodo-L-thyronine sodium salt (T3)	Sigma-Aldrich	Cat# T6397-100MG
Accutase	Thermo Fisher Scientific	Cat# 00-4555-56
Activin A	R&D Systems	Cat# 338-AC/CF
Alk5 Inhibitor II	Cayman Chemicals	Cat# 14794
Bovine Albumin Fraction V	Life Technologies	Cat# 15260037
D-(+)-Glucose Solution, 45%	Sigma-Aldrich	Cat# G8769
GlutaMAX	Thermo Fisher Scientific	Cat# 35050061
Heparin	Sigma-Aldrich	Cat# H3149-250KU
Insulin-Transferrin-Selenium-Ethanolamine (ITS-X)	Thermo Fisher Scientific	Cat# 51500-056
L-Ascorbic Acid	Sigma-Aldrich	Cat# A4544
LDN-193189	Stemgent	Cat# 04-0074

Matrigel®	Corning	Cat# 356231
MCDB 131	Thermo Fisher Scientific	Cat# 10372-019
mTeSR1 Complete Kit - GMP	Stem Cell Technologies	Cat# 85850
Penicillin-Streptomycin	Thermo Fisher Scientific	Cat# 15140122
Recombinant KGF/FGF7	R&D Systems	Cat# 251-KG
Recombinant Mouse Wnt3A	R&D Systems	Cat# 1324-WN/CF
Retinoic Acid	Sigma-Aldrich	Cat# R2625
ROCK Inhibitor Y-27632	Stem Cell Technologies	Cat# 72305
SANT-1	Sigma-Aldrich	Cat# S4572
Sodium Bicarbonate	Sigma-Aldrich	Cat# NC0564699
TPB	Calbiochem	Cat# 565740
Zinc sulfate heptahydrate (ZnSO4)	Sigma-Aldrich	Cat# Z0251

Supplementary Table 3. Short hairpin sequences used for *PDX1* knockdown.

	Sense	Antisense
SCRAM	5'-TGAACAAGATGAAGAGCACCTTCAAGAGAGGTGCTCTTCATCTTGTTCTTTTTTC-3'	5'-TCGAGAAAAAAGAACAAGATGAAGAGCACCTCTCTTGAAGGTGCTCTTCATCTTGTCA-3'
sh*PDX1*	5'-TGGAGTTCCTATTCAACAAGTTCAAGAGACTTGTTGAATAGGAACTCCTTTTTTC-3'	5'-TCGAGAAAAAAGGAGTTCCTATTCAACAAGTCTCTTGAACTTGTTGAATAGGAACTCA-3'

Supplementary Table 4. Primer sequences used for ChIP-qPCR.

Flanking histones #1 forward	AAA GGG GAA GGG AGC ACA TG
Flanking histones #1 reverse	TCC TGC ACA CTG TCT ACT GAT
Flanking histones #2 forward	GCT GGG ATC TCA ATT TGC ATG T
Flanking histones #2 reverse	AGG GAG CAG CTG GTT CTT TA
FOXA binding site #1 forward	AAT TAT AGA TGG TGG TGT CAG GT
FOXA binding site #1 reverse	AAA TTG AGA TCC CAG CCG CC
FOXA binding site #2 forward	GAT GGC ACA CTG ACA GAA GAT
FOXA binding site #2 reverse	GCA GAG TCA ATA GGT GCA GAC
Negative control region forward	TCA AAG GCT CAT CTT TGC AG
Negative control region reverse	AAA GCT GGA CTG GTG AAT GC

Supplementary Table 5. Pearson correlation coefficients of ChIP-seq replicates.

	H3K27ac	H3K4me1	FOXA1	FOXA2	PDX1	HNF6	SOX9	GATA4	GATA6	HNF4A
CyT49 ES	0.94	N/A	N/A	N/A	N/A	N/A	N/A	N/A	N/A	N/A
CyT49 DE	0.91	N/A	N/A	N/A	N/A	N/A	N/A	N/A	N/A	N/A
CyT49 GT	0.88	0.98	0.90	0.90	N/A	N/A	N/A	0.88	0.87	N/A
CyT49 PP1	0.83	N/A	N/A	N/A	N/A	N/A	N/A	N/A	N/A	N/A
CyT49 PP2	0.77	0.99	0.89	0.84	0.64	0.93	0.86	N/A	N/A	N/A
CyT49 HP	0.94	N/A	0.84	0.70	N/A	N/A	N/A	N/A	N/A	0.88
CyT49 PP2 *SCRAM*	0.71	N/A	0.90	0.68	N/A	N/A	N/A	N/A	N/A	N/A
CyT49 PP2 sh*PDX1*	0.86	N/A	0.86	0.62	N/A	N/A	N/A	N/A	N/A	N/A
H1 GT	0.98	0.86	N/A	N/A	N/A	N/A	N/A	N/A	N/A	N/A
H1 PP2	0.97	0.88	N/A	N/A	N/A	N/A	N/A	N/A	N/A	N/A
H1 *FOXA1/2* exon deletion GT	0.98	0.87	N/A	N/A	N/A	N/A	N/A	N/A	N/A	N/A
H1 *FOXA1/2* exon deletion PP2	0.99	0.88	N/A	N/A	N/A	N/A	N/A	N/A	N/A	N/A
iAEC2 DE	1.00	N/A	N/A	N/A	N/A	N/A	N/A	N/A	N/A	N/A
iAEC2 AFG	1.00	N/A	0.99	N/A	N/A	N/A	N/A	N/A	N/A	N/A
iAEC2 ALV	1.00	N/A	0.95	N/A	N/A	N/A	N/A	N/A	N/A	N/A

Supplementary Table 6. Primer sequences used for RT-qPCR.

PDX1 forward	AAG TCT ACC AAA GCT CAC GCG
PDX1 reverse	GTA GGC GCC GCC TGC
NKX6.1 forward	CTG GCC TGT ACC CCT CAT CA
NKX6.1 reverse	CTT CCC GTC TTT GTC CAA CA
FOXA1 forward	GAA GAT GGA AGG GCA TGA AA
FOXA1 reverse	GCC TGA GTT CAT GTT GCT GA
FOXA2 forward	GGG AGC GGT GAA GAT GGA
FOXA2 reverse	TCA TGT TGC TCA CGG AGG AGT A
GCG forward	AAG CAT TTA CTT TGT GGC TGG ATT
GCG reverse	TGA TCT GGA TTT CTC CTC TGT GTC T
TBP forward	TGT GCA CAG GAG CCA AGA GT
TBP reverse	ATT TTC TTG CTG CCA GTC TGG

Supplementary Table 7. Pearson correlation coefficients of RNA-seq replicates.

	Non-transfected control H1 hESCs	Non-edited H1 hESCs	*FOXA1/2* frameshift mutation H1 hESCs	*FOXA1/2* exon deletion H1 hESCs
DE	0.89	0.92	0.92	0.93
GT	0.96	0.91	0.91	0.92
PP1	0.98	0.98	0.93	0.94

PP2	0.99	0.96	0.83	0.90

Supplementary Table 8. Pearson correlation coefficients of ATAC-seq replicates.

	Non-transfected control H1 hESCs	*FOXA1/2* exon deletion H1 hESCs	*SCRAM* transduced CyT49 hESCs	sh*PDX1* transduced CyT49 hESCs
GT	0.99	0.98	N/A	N/A
PP2	0.97	0.99	0.99	1.00
