# Supplementary material for: Sequence logic at enhancers governs a dual mechanism of endodermal organ fate induction by FOXA pioneer factors
Source: Nat Commun. 2021 Nov 17;12:6636. doi: 10.1038/s41467-021-26950-0 (PMC8599738; doi:10.1038/s41467-021-26950-0)
Supplement: Supplementary file 3 — Description of additional Supplementary File [file 41467_2021_26950_MOESM3_ESM.pdf]

Supplementary Data:

Supplementary Data 1. Genes regulated by FOXA1/2 at GT stage. All differential expression was determined using DESeq2.
(supplied as Excel file: Supplementary_Data_1.xlsx)

Supplementary Data 2. Genes regulated by FOXA1/2 at PP2 stage. All differential expression was determined using DESeq2.
(supplied as Excel file: Supplementary_Data_2.xlsx)

Supplementary Data 3. Class I pancreatic enhancers.
(supplied as Excel file: Supplementary_Data_3.xlsx)

Supplementary Data 4. Class II pancreatic enhancers.
(supplied as Excel file: Supplementary_Data_4.xlsx)

Supplementary Data 5. Class III pancreatic enhancers.
(supplied as Excel file: Supplementary_Data_5.xlsx)

Supplementary Data 6. Pancreatic super-enhancers.
(supplied as Excel file: Supplementary_Data_6.xlsx)

Supplementary Data 7. Chromatin loops at PP2 stage.
(supplied as Excel file: Supplementary_Data_7.xlsx)

Supplementary Data 8. Expressed genes in PP2 proximal to class I pancreatic enhancers.
(supplied as Excel file: Supplementary_Data_8.xlsx)

Supplementary Data 9. Expressed genes in PP2 proximal to class II pancreatic enhancers.
(supplied as Excel file: Supplementary_Data_9.xlsx)

Supplementary Data 10. Enriched gene ontology terms of genes associated with class I compared to class II pancreatic enhancers. Enrichment was determined using Metascape.
(supplied as Excel file: Supplementary_Data_10.xlsx)

Supplementary Data 11. *De novo* motifs enriched in class I pancreatic enhancers over class II pancreatic enhancers. Enriched binding motifs were determined using Fisher's exact test, 1 sided, adjusted for multiple comparisons.
(supplied as Excel file: Supplementary_Data_11.xlsx)

Supplementary Data 12. *De novo* motifs enriched in class II pancreatic enhancers over class I pancreatic enhancers. Enriched binding motifs were determined using Fisher's exact test, 1 sided, adjusted for multiple comparisons.
(supplied as Excel file: Supplementary_Data_12.xlsx)

Supplementary Data 13. *De novo* motifs enriched in class I pancreatic enhancers overlapping HNF6 binding sites at PP2 stage compared to class II pancreatic enhancers

overlapping HNF6 binding sites at PP2 stage. Enriched binding motifs were determined using Fisher's exact test, 1 sided, adjusted for multiple comparisons.
(supplied as Excel file: Supplementary_Data_13.xlsx)

Supplementary Data 14. *De novo* motifs enriched in class II pancreatic enhancers overlapping HNF6 binding sites at PP2 stage compared to class I pancreatic enhancers overlapping HNF6 binding sites at PP2 stage. Enriched binding motifs were determined using Fisher's exact test, 1 sided, adjusted for multiple comparisons.
(supplied as Excel file: Supplementary_Data_14.xlsx)

Supplementary Data 15. *De novo* motifs enriched in class I pancreatic enhancers overlapping PDX1 binding sites at PP2 stage compared to class II pancreatic enhancers overlapping PDX1 binding sites at PP2 stage. Enriched binding motifs were determined using Fisher's exact test, 1 sided, adjusted for multiple comparisons.
(supplied as Excel file: Supplementary_Data_15.xlsx)

Supplementary Data 16. *De novo* motifs enriched in class II pancreatic enhancers overlapping PDX1 binding sites at PP2 stage compared to class I pancreatic enhancers overlapping PDX1 binding sites at PP2 stage. Enriched binding motifs were determined using Fisher's exact test, 1 sided, adjusted for multiple comparisons.
(supplied as Excel file: Supplementary_Data_16.xlsx)

Supplementary Data 17. *De novo* motifs enriched in class I pancreatic enhancers overlapping SOX9 binding sites at PP2 stage compared to class II pancreatic enhancers overlapping SOX9 binding sites at PP2 stage. Enriched binding motifs were determined using Fisher's exact test, 1 sided, adjusted for multiple comparisons.
(supplied as Excel file: Supplementary_Data_17.xlsx)

Supplementary Data 18. *De novo* motifs enriched in class II pancreatic enhancers overlapping SOX9 binding sites at PP2 stage compared to class I pancreatic enhancers overlapping SOX9 binding sites at PP2 stage. Enriched binding motifs were determined using Fisher's exact test, 1 sided, adjusted for multiple comparisons.
(supplied as Excel file: Supplementary_Data_18.xlsx)

Supplementary Data 19. Class I hepatic enhancers.
(supplied as Excel file: Supplementary_Data_19.xlsx)

Supplementary Data 20. Class II hepatic enhancers.
(supplied as Excel file: Supplementary_Data_20.xlsx)

Supplementary Data 21. Class III hepatic enhancers.
(supplied as Excel file: Supplementary_Data_21.xlsx)

Supplementary Data 22. Class I alveolar enhancers.
(supplied as Excel file: Supplementary_Data_22.xlsx)

Supplementary Data 23. Class II alveolar enhancers.
(supplied as Excel file: Supplementary_Data_23.xlsx)

Supplementary Data 24. Class III alveolar enhancers.
(supplied as Excel file: Supplementary_Data_24.xlsx)

Supplementary Data 25. ***De novo*** **motifs enriched in class I hepatic enhancers over class II hepatic enhancers. Enriched binding motifs were determined using Fisher's exact test, 1 sided, adjusted for multiple comparisons.**
(supplied as Excel file: Supplementary_Data_25.xlsx)

Supplementary Data 26. ***De novo*** **motifs enriched in class II hepatic enhancers over class I hepatic enhancers. Enriched binding motifs were determined using Fisher's exact test, 1 sided, adjusted for multiple comparisons.**
(supplied as Excel file: Supplementary_Data_26.xlsx)

Supplementary Data 27. ***De novo*** **motifs enriched in class I alveolar enhancers over class II alveolar enhancers. Enriched binding motifs were determined using Fisher's exact test, 1 sided, adjusted for multiple comparisons.**
(supplied as Excel file: Supplementary_Data_27.xlsx)

Supplementary Data 28. ***De novo*** **motifs enriched in class II alveolar enhancers over class I alveolar enhancers. Enriched binding motifs were determined using Fisher's exact test, 1 sided, adjusted for multiple comparisons.**
(supplied as Excel file: Supplementary_Data_28.xlsx)

Supplementary Data 29. ***De novo*** **motifs enriched in class I hepatic enhancers overlapping HNF4A binding sites at HP stage compared to class II hepatic enhancers overlapping HNF4A binding sites at HP stage. Enriched binding motifs were determined using Fisher's exact test, 1 sided, adjusted for multiple comparisons.**
(supplied as Excel file: Supplementary_Data_29.xlsx)

Supplementary Data 30. ***De novo*** **motifs enriched in class II hepatic enhancers overlapping HNF4A binding sites at HP stage compared to class I hepatic enhancers overlapping HNF4A binding sites at HP stage. Enriched binding motifs were determined using Fisher's exact test, 1 sided, adjusted for multiple comparisons.**
(supplied as Excel file: Supplementary_Data_30.xlsx)

Supplementary Data 31. Known motifs enriched in class I pancreatic over class I hepatic and alveolar enhancers. Enriched binding motifs were determined using Fisher's exact test, 1 sided, adjusted for multiple comparisons.
(supplied as Excel file: Supplementary_Data_31.xlsx)

Supplementary Data 32. Known motifs enriched in class I hepatic over class I pancreatic and alveolar enhancers. Enriched binding motifs were determined using Fisher's exact test, 1 sided, adjusted for multiple comparisons.
(supplied as Excel file: Supplementary_Data_32.xlsx)

Supplementary Data 33. Known motifs enriched in class I alveolar over class I pancreatic and hepatic enhancers. Enriched binding motifs were determined using Fisher's exact test, 1 sided, adjusted for multiple comparisons.
(supplied as Excel file: Supplementary_Data_33.xlsx)

Supplementary Data 34. Known motifs enriched in class II pancreatic over class II hepatic and alveolar enhancers. Enriched binding motifs were determined using Fisher's exact test, 1 sided, adjusted for multiple comparisons.

(supplied as Excel file: Supplementary_Data_34.xlsx)

Supplementary Data 35. Known motifs enriched in class II hepatic over class II pancreatic and alveolar enhancers. Enriched binding motifs were determined using Fisher's exact test, 1 sided, adjusted for multiple comparisons.
(supplied as Excel file: Supplementary_Data_35.xlsx)

Supplementary Data 36. Known motifs enriched in class II alveolar over class II pancreatic and hepatic enhancers. Enriched binding motifs were determined using Fisher's exact test, 1 sided, adjusted for multiple comparisons.
(supplied as Excel file: Supplementary_Data_36.xlsx)
