# Supplementary material for: Sequence logic at enhancers governs a dual mechanism of endodermal organ fate induction by FOXA pioneer factors
Source: Nat Commun. 2021 Nov 17;12:6636. doi: 10.1038/s41467-021-26950-0 (PMC8599738; doi:10.1038/s41467-021-26950-0)
Supplement: Supplementary file 40 — Reporting Summary [file 41467_2021_26950_MOESM40_ESM.pdf]

nature research

Corresponding author(s): Maike Sander

Last updated by author(s): Oct 12, 2021

Reporting Summary

Nature Research wishes to improve the reproducibility of the work that we publish. This form provides structure for consistency and transparency in reporting. For further information on Nature Research policies, see our Editorial Policies and the Editorial Policy Checklist.

Statistics

For all statistical analyses, confirm that the following items are present in the figure legend, table legend, main text, or Methods section.

n/a	Confirmed	
☐	☒	The exact sample size (*n*) for each experimental group/condition, given as a discrete number and unit of measurement
☐	☒	A statement on whether measurements were taken from distinct samples or whether the same sample was measured repeatedly
☐	☒	The statistical test(s) used AND whether they are one- or two-sided<br>*Only common tests should be described solely by name; describe more complex techniques in the Methods section.*
☒	☐	A description of all covariates tested
☐	☒	A description of any assumptions or corrections, such as tests of normality and adjustment for multiple comparisons
☐	☒	A full description of the statistical parameters including central tendency (e.g. means) or other basic estimates (e.g. regression coefficient) AND variation (e.g. standard deviation) or associated estimates of uncertainty (e.g. confidence intervals)
☐	☒	For null hypothesis testing, the test statistic (e.g. *F*, *t*, *r*) with confidence intervals, effect sizes, degrees of freedom and *P* value noted<br>*Give P values as exact values whenever suitable.*
☒	☐	For Bayesian analysis, information on the choice of priors and Markov chain Monte Carlo settings
☒	☐	For hierarchical and complex designs, identification of the appropriate level for tests and full reporting of outcomes
☐	☒	Estimates of effect sizes (e.g. Cohen's *d*, Pearson's *r*), indicating how they were calculated

Our web collection on statistics for biologists contains articles on many of the points above.

Software and code

Policy information about availability of computer code

Data collection	No software was used for data collection.

Data analysis	Burrows-Wheeler Aligner v 0.7.13 was used for mapping of ChIP-seq and ATAC-seq data to the human reference genome hg19 with default parameters.<br>STAR v2.4.0f1 was used to map RNA-seq data to the human reference genome hg19 with default parameters.<br>Samtools v1.5 was used to remove reads marked as duplicates with default parameters.<br>The Bedtools suite v2.17.0 was used to perform genomic algebra operations.<br>The HOMER suite of bioinformatics tools were used to call ChIP-seq peaks, annotate peaks, perform differential peak and gene expression (invoking the R program DESeq2 v3.10) analysis, and generate bigWig files for data visualization. All programs were run using default settings. Default FDR for HOMER ChIP-seq peak calling is ≤ 0.001.<br>Cufflinks v2.2.1 was used to calculate fpkms for RNA-seq datasets with the parameters: --library-type fr-firststrand --max-bundle-frags 10000000<br>MACS2 v2.1.4 was used to call ATAC-seq peaks, with parameters "shift set to 100 bps, smoothing window of 200 bps" and with "nolambda" and "nomodel" flags on. MACS2 was also used to call ATAC-Seq summits, using the same parameters combined with the "call-summits" flag.<br>Correlations between datasets were calculated using deepTools2 v3.1.3<br>Gene ontology analysis for enhancer groups was performed using GREAT v4.0.4 with the default parameters. Gene ontology for differentially expressed genes was performed using Metascape using default parameters.<br>Super-enhancers were defined using the Rank Order Super-Enhancers (ROSE) package using default parameters (no version number). Specifically, pancreatic enhancers were ranked based on PP2 H3K27ac signal and super-enhancers were defined as enhancers ranked beyond the inflection point.<br>All scRNA-seq analyses were performed using Seurat version 3.0.<br>FlowJo v10 software was used to analyze flow cytometry data.

For manuscripts utilizing custom algorithms or software that are central to the research but not yet described in published literature, software must be made available to editors and reviewers. We strongly encourage code deposition in a community repository (e.g. GitHub). See the Nature Research guidelines for submitting code & software for further information.

Data

Policy information about availability of data

All manuscripts must include a data availability statement. This statement should provide the following information, where applicable:

- Accession codes, unique identifiers, or web links for publicly available datasets
- A list of figures that have associated raw data
- A description of any restrictions on data availability

The following datasets used in this study were obtained from the GEO and ArrayExpress repositories:
RNA-seq: Pancreatic differentiation of CyT49 hESC line (E-MTAB-1086 https://www.ebi.ac.uk/arrayexpress/experiments/E-MTAB-1086/)
ChIP-seq: H3K27ac in CyT49 hESC, DE, GT, PP1, PP2 (GSE54471 https://www.ncbi.nlm.nih.gov/geo/query/acc.cgi?acc=GSE54471 and GSE149148 https://www.ncbi.nlm.nih.gov/geo/query/acc.cgi?acc=GSE149148); H3K27ac in CyT49 PP2 SCRAM and PP2 shPDX1 (GSE54471 https://www.ncbi.nlm.nih.gov/geo/query/acc.cgi?acc=GSE54471); H3K4me1 in CyT49 GT and PP2 (GSE54471 https://www.ncbi.nlm.nih.gov/geo/query/acc.cgi?acc=GSE54471 and GSE149148 https://www.ncbi.nlm.nih.gov/geo/query/acc.cgi?acc=GSE149148); RXR in CyT49 PP1 (GSE104840 https://www.ncbi.nlm.nih.gov/geo/query/acc.cgi?acc=GSE104840); PDX1 in CyT49 PP2 (GSE54471 https://www.ncbi.nlm.nih.gov/geo/query/acc.cgi?acc=GSE54471 and GSE149148 https://www.ncbi.nlm.nih.gov/geo/query/acc.cgi?acc=GSE149148); HNF6 in CyT49 PP2 (GSE149148 https://www.ncbi.nlm.nih.gov/geo/query/acc.cgi?acc=GSE149148); SOX9 in CyT49 PP2 (GSE149148 https://www.ncbi.nlm.nih.gov/geo/query/acc.cgi?acc=GSE149148); FOXA1 in CyT49 PP2 (GSE149148 https://www.ncbi.nlm.nih.gov/geo/query/acc.cgi?acc=GSE149148); FOXA2 in CyT49 PP2 (GSE149148 https://www.ncbi.nlm.nih.gov/geo/query/acc.cgi?acc=GSE149148).
ATAC-seq: CyT49 GT and PP2 (GSE149148 https://www.ncbi.nlm.nih.gov/geo/query/acc.cgi?acc=GSE149148)
Hi-C datasets were generated as a component of the 4D Nucleome Project78. Datasets corresponding to the PP2 stages of differentiation can be found under accession number 4DNESOLVRKBM https://data.4dnucleome.org/experiment-set-replicates/4DNESOLVRKBM/.

All mRNA-seq, ChIP-seq, and ATAC-seq datasets generated for this study have been deposited at GEO under the accession number GSE148368 https://www.ncbi.nlm.nih.gov/geo/query/acc.cgi?acc=GSE148368. The source data underlying Figs. 1b, c, d, e, 6b, e, and Supplementary Figs. 1f, g, 5c, 6a, 7d, g, and 9a are provided as a Source Data file.

Field-specific reporting

Please select the one below that is the best fit for your research. If you are not sure, read the appropriate sections before making your selection.

- [x] Life sciences
- [ ] Behavioural & social sciences
- [ ] Ecological, evolutionary & environmental sciences

For a reference copy of the document with all sections, see nature.com/documents/nr-reporting-summary-flat.pdf

Life sciences study design

All studies must disclose on these points even when the disclosure is negative.

Sample size	No sample size calculations were performed. Two to three biological replicates for ChIP-seq and RNA-seq was determined to be sufficient based on the research community standards when the studies were performed. https://www.encodeproject.org/about/experiment-guidelines
Data exclusions	Only data derived from failed differentiations, as measured by IFC, qPCR and flow cytometry for successful generation of pancreatic progenitor cells in control samples, was excluded as this data is not representative of a valid differentiation.

Replication	The experiment differentiating FOXA1/2 -/- hESCs was repeated > 10 times and always resulted in the same phenotype showing a complete lack of pancreatic progenitor cells. The experiments differentiating FOXA1 -/- and FOXA2 -/- hESCs as well as shPDX1 and shSCRAM hESCs were repeated at least 3 times, showing a consistent phenotype for each differentiation. The experiment differentiating motif optimized hESCs was repeated at least 5 times, showing a consistent phenotype for each differentiation. For NGS sequencing data from biological replicates the correlation coefficient between replicates was calculated: Pearson correlation ≥0.7 for ChIP-seq data; Pearson correlation ≥0.89 for RNA-seq data; Pearson correlation ≥.97 for ATAC-seq data.
Randomization	Randomization was not necessary because cells in each experiment were assayed at specific maturation time points or in the context of specific genetic modifications.
Blinding	Blinding was not performed because values derived from all experiments were quantitative and did not require subjective interpretation.

Reporting for specific materials, systems and methods

We require information from authors about some types of materials, experimental systems and methods used in many studies. Here, indicate whether each material, system or method listed is relevant to your study. If you are not sure if a list item applies to your research, read the appropriate section before selecting a response.

Materials & experimental systems

n/a	Involved in the study	
☐	☒	Antibodies
☐	☒	Eukaryotic cell lines
☒	☐	Palaeontology and archaeology
☒	☐	Animals and other organisms
☒	☐	Human research participants
☒	☐	Clinical data
☒	☐	Dual use research of concern

Methods

n/a	Involved in the study	
☐	☒	ChIP-seq
☐	☒	Flow cytometry
☒	☐	MRI-based neuroimaging

Antibodies

Antibodies used

Primary IFC Staining:
mouse anti-FOXA1 Abcam Cat# ab55178, RRID:AB_941631
goat anti-FOXA2 R&D Systems Cat# AF2400, RRID:AB_2294104
goat anti-SOX17 R&D Systems Cat# AF1924, RRID:AB_355060
goat anti-HNF4A Santa Cruz Biotechnology Cat# SC-6556, RRID:AB_2117025
rabbit anti-PDX1 Abcam Cat# ab47267, RRID:AB_777179
mouse anti-NKX6.1 Developmental Studies Hybridoma Bank Cat# F64A6B4, RRID:AB_532380

Secondary IFC Staining:
Cy3-conjugated donkey anti-mouse Jackson ImmunoResearch Labs Cat# 715-165-150, RRID:AB_2340813
Alexa488-conjugated donkey anti-goat Jackson ImmunoResearch Labs Cat# 705-545-003, RRID:AB_2340428
Alexa488-conjugated donkey anti-rabbit Jackson ImmunoResearch Labs Cat# 711-485-152, RRID:AB_2492289

Flow Cytometry:
APC Mouse IgG1, κ Isotype Control BD Pharmingen Cat# 555751, RRID:AB_398613
mouse anti-HNF1B Santa Cruz Biotechnology Cat# sc-130407, RRID:AB_2248215
mouse anti-NKX6.1-Alexa Fluor® 647 BD Biosciences Cat# 563338, RRID:AB_2738144
mouse anti-PDX1-PE BD Biosciences Cat# 562161, RRID:AB_10893589
mouse anti-SOX17-PE BD Biosciences Cat# 561591, RRID:AB_10717121
rabbit anti-Insulin-PE Cell Signaling Cat # 8508, RRID:AB_11179076
PE Mouse IgG1, κ Isotype Control BD Pharmingen Cat# 555749, RRID:AB_396091

ChIP-seq:
goat anti-FOXA1 Abcam Cat# ab5089, RRID:AB_304744
goat anti-FOXA2 Santa Cruz Biotechnology Cat# sc-6554, RRID:AB_2262810
mouse anti-HNF4A Novus Cat # PP-H1415, RRID:AB_1964276
rabbit anti-H3K27ac Active Motif Cat# 39133, RRID:AB_2561016
rabbit anti-H3K4me1 Abcam Cat# ab8895, RRID:AB_306847
goat anti-GATA4 Santa Cruz Biotechnology Cat# sc-1237, RRID:AB_2108747
mouse anti-GATA6 Santa Cruz Biotechnology Cat# sc-9055, RRID:AB_2108768

ChIP-qPCR:
goat anti-FOXA1 Abcam Cat# ab5089, RRID:AB_304744
goat anti-FOXA2 R&D Cat# AF2400, RRID:AB_2294104
rabbit anti-H3K27ac Active Motif Cat# 39133, RRID:AB_2561016

rabbit anti-H3K4me1 Abcam Cat# ab8895, RRID:AB_306847

Validation

Primary IFC Staining:
goat anti-PDX1 Abcam Cat# ab47383, RRID:AB_2162359
The manufacturer's website shows validation that the antibody is specific for both mouse and human PDX1 protein. The antibody is highly cited for IFC according to citeab.com.

mouse anti-NKX6.1 Developmental Studies Hybridoma Bank Cat# F64A6B4, RRID:AB_532380
The manufacturer's website shows validation that the antibody is specific for both mouse and human NKX6.1 protein. The antibody is highly cited for IFC according to citeab.com.

goat anti-SOX17 R&D Systems Cat# AF1924, RRID:AB_355060
The manufacturer's website shows validation that the antibody is specific for both mouse and human SOX17 protein. The antibody is highly cited for IFC according to citeab.com.

goat anti-HNF4A Santa Cruz Biotechnology Cat# SC-6556, RRID:AB_2117025
The manufacture's website shows validation that the antibody is specific for human HNF4A protein. The antibody is highly cited for IFC according to the manufacturer's website.

mouse anti-FOXA1 Abcam Cat# ab55178, RRID:AB_941631
The manufacture's website shows validation that the antibody is specific for human FOXA1 protein. The antibody is highly cited for IFC according to citeab.com.

goat anti-FOXA2 R&D Systems Cat# AF2400, RRID:AB_2294104
The manufacture's website shows validation that the antibody is specific for human FOXA2 protein. The antibody is highly cited for IFC according to the manufacturer's website.

Secondary IFC Staining:
Cy3-conjugated donkey anti-mouse Jackson ImmunoResearch Labs Cat# 715-165-150, RRID:AB 2340813
The manufacturer's website shows validation that the antibody is specific for mouse IgG protein. The antibody is highly cited for IFC according to citeab.com.

Alexa488-conjugated donkey anti-goat Jackson ImmunoResearch Labs Cat# 705-545-003, RRID:AB_2340428
The manufacturer's website shows validation that the antibody is specific for goat IgG protein. The antibody is highly cited for IFC according to citeab.com.

Alexa488-conjugated donkey anti-rabbit Jackson ImmunoResearch Labs Cat# 711-485-152, RRID:AB_2492289
The manufacturer's website shows validation that the antibody is specific for rabbit IgG protein. The antibody is highly cited for IFC based on its RRID.

Flow Cytometry:
APC Mouse IgG1, κ Isotype Control BD Pharmingen Cat# 555751, RRID:AB_398613
The manufacturer's website lists that the antibody is specific for mouse MOPC-21 IgG protein. The antibody is highly cited for flow cytometry according to citeab.com.

mouse anti-NKX6.1-Alexa Fluor® 647 BD Biosciences Cat# 563338, RRID:AB_2738144
The manufacture's website shows validation that the antibody is specific for both mouse and human NKX6.1 protein and cites several studies in which the antibody was used for flow cytometry.

mouse anti-PDX1-PE BD Biosciences Cat# 562161, RRID:AB_10893589
The manufacture's website shows validation that the antibody is specific for both mouse and human PDX1 protein and cites several studies in which the antibody was used for flow cytometry.

mouse anti-HNF1B Santa Cruz Biotechnology Cat# sc-130407, RRID:AB_2248215
The manufacture's website shows validation that the antibody is specific for human HNF1B protein and the antibody was used for flow cytometry in a recent study (PMID: 31291575).

mouse anti-SOX17-PE BD Biosciences Cat# 561591, RRID:AB_10717121
The manufacture's website shows validation that the antibody is specific for both mouse and human SOX17 protein and cites several studies in which the antibody was used for flow cytometry.

rabbit anti-Insulin-PE Cell Signaling Cat # 8508, RRID:AB_11179076
The manufacture's website shows validation that the antibody is specific for both mouse and human Insulin protein and cites several studies in which the antibody was used for flow cytometry.

PE Mouse IgG1, κ Isotype Control BD Pharmingen Cat# 555749, RRID:AB_396091
The manufacturer's website lists that the antibody is specific for mouse MOPC-21 IgG protein. The antibody is highly cited for flow cytometry according to citeab.com.

ChIP-seq:

rabbit anti-H3K27ac Active Motif Cat# 39133, RRID:AB_2561016
The manufacture's website shows validation that the antibody is specific for human H3K27ac protein. The antibody is highly cited for ChIP according to citeab.com and was validated by ENCODE.

rabbit anti-H3K4me1 Abcam Cat# ab8895, RRID:AB_306847
The manufacture's website shows validation that the antibody is specific for human H3K4me1 protein. The antibody is highly cited for ChIP according to citeab.com and was validated by ENCODE. Also recently rigorously evaluated for target specificity in PMID: 30244833.

goat anti-FOXA1 Abcam Cat# ab5089, RRID:AB_304744
The manufacture's website shows validation that the antibody is specific for both mouse and human FOXA1 protein. The antibody is highly cited for ChIP according to citeab.com.

goat anti-FOXA2 Santa Cruz Biotechnology Cat# sc-6554, RRID:AB_2262810
The manufacture's website shows validation that the antibody is specific for both mouse and human FOXA2 protein. The antibody is highly cited for ChIP and is being validated by ENCODE.

mouse anti-HNF4A Novus Cat # PP-H1415, RRID:AB_1964276
The manufacture's website shows validation that the antibody is specific for both mouse and human HNF4A protein. The antibody is cited for ChIP according to citeab.com.

goat anti-GATA4 Santa Cruz Biotechnology Cat# sc-1237, RRID:AB_2108747
The manufacture's website shows validation that the antibody is specific for both mouse and human GATA4 protein. The antibody is highly cited for ChIP.

mouse anti-GATA6 Santa Cruz Biotechnology Cat# sc-9055, RRID:AB_2108768
The manufacture's website shows validation that the antibody is specific for both mouse and human GATA6 protein. The antibody has been validated in PMID:30982595.

ChIP-qPCR:
rabbit anti-H3K27ac Active Motif Cat# 39133, RRID:AB_2561016
The manufacture's website shows validation that the antibody is specific for human H3K27ac protein. The antibody is highly cited for ChIP according to citeab.com and was validated by ENCODE.

rabbit anti-H3K4me1 Abcam Cat# ab8895, RRID:AB_306847
The manufacture's website shows validation that the antibody is specific for human H3K4me1 protein. The antibody is highly cited for ChIP according to citeab.com and was validated by ENCODE. Also recently rigorously evaluated for target specificity in PMID: 30244833.

goat anti-FOXA1 Abcam Cat# ab5089, RRID:AB_304744
The manufacture's website shows validation that the antibody is specific for both mouse and human FOXA1 protein. The antibody is highly cited for ChIP according to citeab.com.

goat anti-FOXA2 R&D Cat# AF2400, RRID:AB_2294104
The manufacture's website shows validation that the antibody is specific for human FOXA2 protein. The antibody is highly cited fro ChIP and has been validated in PMID:31291575.

Eukaryotic cell lines

Policy information about cell lines

Cell line source(s)	CyT49 hESCs, ViaCyte, Inc., NIHhESC-10-0041, RRID:CVCL_B850<br>H1 hESCs, WiCell Research Institute, NIHhESC-10-0043, RRID:CVCL_9771<br>SPC2-ST-B2 hiPSC line, Kotton Lab (Hurley, et al. 2020), http://www.bumc.bu.edu/stemcells<br>HEK293T, ATCC, Cat# CRL-3216, RRID:CVCL_0063
Authentication	All cell experiments were conducted on batches of cryopreserved cells that were tested for mycoplasma and confirmed negative at the time of freezing. hESCs (CyT49, H1, and SPC2-ST-B2) were karyotyped to ensure genomic stability. In each experiment, proper differentiation of CyT49 and H1 hESCs towards the pancreatic fate was validated by flow cytometry analysis for stage-specific markers at the definitive endoderm, gut tube, and pancreatic progenitor stages. Differentiation of CyT49 cells towards the hepatic fate was validated by flow cytometry analysis for stage-specific markers at the definitive endoderm, gut tube, and hepatic progenitor stages. SPC2-ST-B2 hiPSCs differentiated into alveolospheres were tested for culture quality and purity at each passage using flow cytometry. HEK293T cells were purchased from ATCC. The company validates cell line identity through STR analysis.
Mycoplasma contamination	Cell lines were tested for mycoplasma on a quarterly basis. No positive results were found.
Commonly misidentified lines<br>(See ICLAC register)	No commonly misidentified cell lines were used in the study.

ChIP-seq

Data deposition

- [x] Confirm that both raw and final processed data have been deposited in a public database such as GEO.
- [x] Confirm that you have deposited or provided access to graph files (e.g. BED files) for the called peaks.

Data access links
May remain private before publication.

To review GEO accession GSE148368:
Go to https://www.ncbi.nlm.nih.gov/geo/query/acc.cgi?acc=GSE148368.

Files in database submission

GSM4462965 Cyt49_GT.ChIP-Seq.FOXA1.replicate.1
GSM4462966 Cyt49_GT.ChIP-Seq.FOXA1.replicate.2
GSM4462967 Cyt49_GT.ChIP-Seq.FOXA2.replicate.1
GSM4462968 Cyt49_GT.ChIP-Seq.FOXA2.replicate.2
GSM4462976 Cyt49_HP.ChIP-Seq.FOXA1.replicate.1
GSM4462977 Cyt49_HP.ChIP-Seq.FOXA1.replicate.2
GSM4462978 Cyt49_HP.ChIP-Seq.FOXA2.replicate.1
GSM4462979 Cyt49_HP.ChIP-Seq.FOXA2.replicate.2
GSM4462984 Cyt49_HP.ChIP-Seq.H3K27ac.replicate.1
GSM4462985 Cyt49_HP.ChIP-Seq.H3K27ac.replicate.2
GSM4462986 Cyt49_HP.ChIP-Seq.HNF4A.replicate.1
GSM4462987 Cyt49_HP.ChIP-Seq.HNF4A.replicate.2
GSM4462988 Cyt49_HP.ChIP-Seq.Input.replicate.1
GSM4463005 Cyt49_PP2_shPDX1.ChIP-Seq.FOXA1.replicate.1
GSM4463006 Cyt49_PP2_shPDX1.ChIP-Seq.FOXA1.replicate.2
GSM4463007 Cyt49_PP2_shPDX1.ChIP-Seq.FOXA2.replicate.1
GSM4463008 Cyt49_PP2_shPDX1.ChIP-Seq.FOXA2.replicate.2
GSM4463009 Cyt49_PP2_shPDX1.ChIP-Seq.Input.replicate.1
GSM4463010 Cyt49_PP2_shSCRAM.ChIP-Seq.FOXA1.replicate.1
GSM4463011 Cyt49_PP2_shSCRAM.ChIP-Seq.FOXA1.replicate.2
GSM4463012 Cyt49_PP2_shSCRAM.ChIP-Seq.FOXA2.replicate.1
GSM4463013 Cyt49_PP2_shSCRAM.ChIP-Seq.FOXA2.replicate.2
GSM4463014 Cyt49_PP2_shSCRAM.ChIP-Seq.Input.replicate.1
GSM4463015 H1_FOXA1_2_Exon_Deletion_GT.ChIP-Seq.H3K27ac.replicate.1
GSM4463016 H1_FOXA1_2_Exon_Deletion_GT.ChIP-Seq.H3K27ac.replicate.2
GSM4463017 H1_FOXA1_2_Exon_Deletion_GT.ChIP-Seq.H3K4me1.replicate.1
GSM4463018 H1_FOXA1_2_Exon_Deletion_GT.ChIP-Seq.H3K4me1.replicate.2
GSM4463019 H1_FOXA1_2_Exon_Deletion_GT.ChIP-Seq.Input.replicate.1
GSM4463020 H1_FOXA1_2_Exon_Deletion_PP2.ChIP-Seq.H3K27ac.replicate.1
GSM4463021 H1_FOXA1_2_Exon_Deletion_PP2.ChIP-Seq.H3K27ac.replicate.2
GSM4463022 H1_FOXA1_2_Exon_Deletion_PP2.ChIP-Seq.H3K4me1.replicate.1
GSM4463023 H1_FOXA1_2_Exon_Deletion_PP2.ChIP-Seq.H3K4me1.replicate.2
GSM4463024 H1_FOXA1_2_Exon_Deletion_PP2.ChIP-Seq.Input.replicate.1
GSM4463025 H1_WT_GT.ChIP-Seq.H3K27ac.replicate.1
GSM4463026 H1_WT_GT.ChIP-Seq.H3K27ac.replicate.2
GSM4463027 H1_WT_GT.ChIP-Seq.H3K4me1.replicate.1
GSM4463028 H1_WT_GT.ChIP-Seq.H3K4me1.replicate.2
GSM4463029 H1_WT_GT.ChIP-Seq.Input.replicate.1
GSM4463030 H1_WT_PP2.ChIP-Seq.H3K27ac.replicate.1
GSM4463031 H1_WT_PP2.ChIP-Seq.H3K27ac.replicate.2
GSM4463032 H1_WT_PP2.ChIP-Seq.H3K4me1.replicate.1
GSM4463033 H1_WT_PP2.ChIP-Seq.H3K4me1.replicate.2
GSM4463034 H1_WT_PP2.ChIP-Seq.Input.replicate.1
GSM4463035 iAEC2_AFG.ChIP-Seq.FOXA1.replicate.1
GSM4463036 iAEC2_AFG.ChIP-Seq.FOXA1.replicate.2
GSM4463037 iAEC2_AFG.ChIP-Seq.H3K27ac.replicate.1
GSM4463038 iAEC2_AFG.ChIP-Seq.H3K27ac.replicate.2
GSM4463039 iAEC2_AFG.ChIP-Seq.Input.replicate.1
GSM4463040 iAEC2_ALV.ChIP-Seq.FOXA1.replicate.1
GSM4463041 iAEC2_ALV.ChIP-Seq.FOXA1.replicate.2
GSM4463042 iAEC2_ALV.ChIP-Seq.H3K27ac.replicate.1
GSM4463043 iAEC2_ALV.ChIP-Seq.H3K27ac.replicate.2
GSM4463044 iAEC2_ALV.ChIP-Seq.Input.replicate.1
GSM4463045 iAEC2_DE.ChIP-Seq.H3K27ac.replicate.1
GSM4463046 iAEC2_DE.ChIP-Seq.H3K27ac.replicate.2
GSM4463047 iAEC2_DE.ChIP-Seq.Input.replicate.1
GSM4750127 Cyt49_PP2_shSCRAM.ChIP-Seq.H3K27ac.replicate.1

GSM4750128 Cyt49_PP2_shPDX1.ChIP-Seq.H3K27ac.replicate.1
Cyt49_GT.ChIP-Seq.GATA4.replicate.1
Cyt49_GT.ChIP-Seq.GATA4.replicate.2
Cyt49_GT.ChIP-Seq.GATA6.replicate.1
Cyt49_GT.ChIP-Seq.GATA6.replicate.2
H1_WT_PP2_scRNA
H1_Motif_Optimized_PP2_scRNA

Genome browser session
(e.g. UCSC)

http://genome.ucsc.edu/cgi-bin/hgTracks?db=hg19&lastVirtModeType=default&lastVirtModeExtraState=&virtModeType=default&virtMode=0&nonVirtPosition=&position=chr4%3A85169312%2D85664511&hgsid=953479511_ZtPnBZdAtKp6GZ4cE6DvaAMA0VHd

Methodology

Replicates

Two biological replicates were used for ChIP-seq data analysis. Replicates were correlated using Pearson correlation (see Methods for correlation table).

Sequencing depth

sample: Cyt49_GT.ChIP-Seq.FOXA1.replicate.1.fastq.gz, total number of reads: 43025575, uniquely mapped reads: 36867973, read length: 50bp, type: single-end
sample: Cyt49_GT.ChIP-Seq.FOXA1.replicate.2.fastq.gz, total number of reads: 34733465, uniquely mapped reads: 27212557, read length: 75bp, type: single-end
sample: Cyt49_GT.ChIP-Seq.FOXA2.replicate.1.fastq.gz, total number of reads: 34339282, uniquely mapped reads: 28842958, read length: 50bp, type: single-end
sample: Cyt49_GT.ChIP-Seq.FOXA2.replicate.2.fastq.gz, total number of reads: 31845004, uniquely mapped reads: 25795234, read length: 75bp, type: single-end
sample: Cyt49_HP.ChIP-Seq.FOXA1.replicate.1.fastq.gz, total number of reads: 22239699, uniquely mapped reads: 17104263, read length: 50bp, type: single-end
sample: Cyt49_HP.ChIP-Seq.FOXA1.replicate.2.fastq.gz, total number of reads: 41956911, uniquely mapped reads: 30352714, read length: 50bp, type: single-end
sample: Cyt49_HP.ChIP-Seq.FOXA2.replicate.1.fastq.gz, total number of reads: 17761357, uniquely mapped reads: 12682084, read length: 50bp, type: single-end
sample: Cyt49_HP.ChIP-Seq.FOXA2.replicate.2.fastq.gz, total number of reads: 155977101, uniquely mapped reads: 118544239, read length: 50bp, type: single-end
sample: Cyt49_HP.ChIP-Seq.H3K27ac.replicate.1.fastq.gz, total number of reads: 40255113, uniquely mapped reads: 34873004, read length: 50bp, type: single-end
sample: Cyt49_HP.ChIP-Seq.H3K27ac.replicate.2.fastq.gz, total number of reads: 38708013, uniquely mapped reads: 33725513, read length: 50bp, type: single-end
sample: Cyt49_HP.ChIP-Seq.HNF4A.replicate.1.fastq.gz, total number of reads: 44213367, uniquely mapped reads: 37625575, read length: 75bp, type: single-end
sample: Cyt49_HP.ChIP-Seq.HNF4A.replicate.2.fastq.gz, total number of reads: 31513636, uniquely mapped reads: 28942123, read length: 75bp, type: single-end
sample: Cyt49_HP.ChIP-Seq.Input.replicate.1.fastq.gz, total number of reads: 20830826, uniquely mapped reads: 12025043, read length: 50bp, type: single-end
sample: Cyt49_PP2_shPDX1.ChIP-Seq.FOXA1.replicate.1.fastq.gz, total number of reads: 63167257, uniquely mapped reads: 58865567, read length: 75bp, type: single-end
sample: Cyt49_PP2_shPDX1.ChIP-Seq.FOXA1.replicate.2.fastq.gz, total number of reads: 38105502, uniquely mapped reads: 33513789, read length: 50bp, type: single-end
sample: Cyt49_PP2_shPDX1.ChIP-Seq.FOXA2.replicate.1.fastq.gz, total number of reads: 49260865, uniquely mapped reads: 42106319, read length: 50bp, type: single-end
sample: Cyt49_PP2_shPDX1.ChIP-Seq.FOXA2.replicate.2.fastq.gz, total number of reads: 19613099, uniquely mapped reads: 15125593, read length: 50bp, type: single-end
sample: Cyt49_PP2_shPDX1.ChIP-Seq.Input.replicate.1.fastq.gz, total number of reads: 19129974, uniquely mapped reads: 12327502, read length: 50bp, type: single-end
sample: Cyt49_PP2_shSCRAM.ChIP-Seq.FOXA1.replicate.1.fastq.gz, total number of reads: 20631615, uniquely mapped reads: 18,450,853, read length: 75bp, type: single-end
sample: Cyt49_PP2_shSCRAM.ChIP-Seq.FOXA1.replicate.2.fastq.gz, total number of reads: 48209818, uniquely mapped reads: 44603724, read length: 75bp, type: single-end
sample: Cyt49_PP2_shSCRAM.ChIP-Seq.FOXA2.replicate.1.fastq.gz, total number of reads: 40217012, uniquely mapped reads: 35076699, read length: 50bp, type: single-end
sample: Cyt49_PP2_shSCRAM.ChIP-Seq.FOXA2.replicate.2.fastq.gz, total number of reads: 18700227, uniquely mapped reads: 14529252, read length: 50bp, type: single-end
sample: Cyt49_PP2_shSCRAM.ChIP-Seq.Input.replicate.1.fastq.gz, total number of reads: 22127410, uniquely mapped reads: 14233517, read length: 50bp, type: single-end
sample: H1_FOXA1_2_Exon_Deletion_GT.ChIP-Seq.H3K27ac.replicate.1.fastq.gz, total number of reads: 41619356, uniquely mapped reads: 37422274, read length: 75bp, type: single-end
sample: H1_FOXA1_2_Exon_Deletion_GT.ChIP-Seq.H3K27ac.replicate.2.fastq.gz, total number of reads: 40630700, uniquely mapped reads: 34418708, read length: 75bp, type: single-end
sample: H1_FOXA1_2_Exon_Deletion_GT.ChIP-Seq.H3K4me1.replicate.1.fastq.gz, total number of reads: 29350526, uniquely mapped reads: 26454325, read length: 75bp, type: single-end
sample: H1_FOXA1_2_Exon_Deletion_GT.ChIP-Seq.H3K4me1.replicate.2.fastq.gz, total number of reads: 41962044, uniquely mapped reads: 35730374, read length: 75bp, type: single-end
sample: H1_FOXA1_2_Exon_Deletion_GT.ChIP-Seq.Input.replicate.1.fastq.gz, total number of reads: 33941103, uniquely mapped

reads: 26298886, read length: 75bp, type: single-end
sample: H1_FOXA1_2_Exon_Deletion_PP2.ChIP-Seq.H3K27ac.replicate.1.fastq.gz, total number of reads: 61657048, uniquely mapped reads: 55997906, read length: 75bp, type: single-end
sample: H1_FOXA1_2_Exon_Deletion_PP2.ChIP-Seq.H3K27ac.replicate.2.fastq.gz, total number of reads: 45740370, uniquely mapped reads: 39791446, read length: 75bp, type: single-end
sample: H1_FOXA1_2_Exon_Deletion_PP2.ChIP-Seq.H3K4me1.replicate.1.fastq.gz, total number of reads: 47302427, uniquely mapped reads: 42860652, read length: 75bp, type: single-end
sample: H1_FOXA1_2_Exon_Deletion_PP2.ChIP-Seq.H3K4me1.replicate.2.fastq.gz, total number of reads: 29915352, uniquely mapped reads: 25768472, read length: 75bp, type: single-end
sample: H1_FOXA1_2_Exon_Deletion_PP2.ChIP-Seq.Input.replicate.1.fastq.gz, total number of reads: 24884485, uniquely mapped reads: 19160186, read length: 75bp, type: single-end
sample: H1_WT_GT.ChIP-Seq.H3K27ac.replicate.1.fastq.gz, total number of reads: 45993590, uniquely mapped reads: 41473794, read length: 75bp, type: single-end
sample: H1_WT_GT.ChIP-Seq.H3K27ac.replicate.2.fastq.gz, total number of reads: 43764022, uniquely mapped reads: 38283346, read length: 75bp, type: single-end
sample: H1_WT_GT.ChIP-Seq.H3K4me1.replicate.1.fastq.gz, total number of reads: 39591376, uniquely mapped reads: 35356985, read length: 75bp, type: single-end
sample: H1_WT_GT.ChIP-Seq.H3K4me1.replicate.2.fastq.gz, total number of reads: 30816030, uniquely mapped reads: 26673110, read length: 75bp, type: single-end
sample: H1_WT_GT.ChIP-Seq.Input.replicate.1.fastq.gz, total number of reads: 26146649, uniquely mapped reads: 20015169, read length: 75bp, type: single-end
sample: H1_WT_PP2.ChIP-Seq.H3K27ac.replicate.1.fastq.gz, total number of reads: 78608981, uniquely mapped reads: 71981543, read length: 75bp, type: single-end
sample: H1_WT_PP2.ChIP-Seq.H3K27ac.replicate.2.fastq.gz, total number of reads: 42313465, uniquely mapped reads: 37169452, read length: 75bp, type: single-end
sample: H1_WT_PP2.ChIP-Seq.H3K4me1.replicate.1.fastq.gz, total number of reads: 32619355, uniquely mapped reads: 29316500, read length: 75bp, type: single-end
sample: H1_WT_PP2.ChIP-Seq.H3K4me1.replicate.2.fastq.gz, total number of reads: 41049132, uniquely mapped reads: 35663193, read length: 75bp, type: single-end
sample: H1_WT_PP2.ChIP-Seq.Input.replicate.1.fastq.gz, total number of reads: 13743037, uniquely mapped reads: 10294979, read length: 75bp, type: single-end
sample: iAEC2_ALV.ChIP-Seq.FOXA1.replicate.1.fastq.gz, total number of reads: 45239562, uniquely mapped reads: 39516757, read length: 75bp, type: single-end
sample: iAEC2_ALV.ChIP-Seq.FOXA1.replicate.2.fastq.gz, total number of reads: 36129230, uniquely mapped reads: 32306757, read length: 75bp, type: single-end
sample: iAEC2_ALV.ChIP-Seq.H3K27ac.replicate.1.fastq.gz, total number of reads: 36249376, uniquely mapped reads: 34552905, read length: 75bp, type: single-end
sample: iAEC2_ALV.ChIP-Seq.H3K27ac.replicate.2.fastq.gz, total number of reads: 35637264, uniquely mapped reads: 33666523, read length: 75bp, type: single-end
sample: iAEC2_ALV.ChIP-Seq.Input.replicate.1.fastq.gz, total number of reads: 18966380, uniquely mapped reads: 18133756, read length: 75bp, type: single-end
sample: iAEC2_AFG.ChIP-Seq.FOXA1.replicate.1.fastq.gz, total number of reads: 72741061, uniquely mapped reads: 64186712, read length: 75bp, type: single-end
sample: iAEC2_AFG.ChIP-Seq.FOXA1.replicate.2.fastq.gz, total number of reads: 68240251, uniquely mapped reads: 62494422, read length: 75bp, type: single-end
sample: iAEC2_AFG.ChIP-Seq.H3K27ac.replicate.1.fastq.gz, total number of reads: 64630028, uniquely mapped reads: 61954345, read length: 75bp, type: single-end
sample: iAEC2_AFG.ChIP-Seq.H3K27ac.replicate.2.fastq.gz, total number of reads: 64006411, uniquely mapped reads: 60345244, read length: 75bp, type: single-end
sample: iAEC2_AFG.ChIP-Seq.Input.replicate.1.fastq.gz, total number of reads: 62086476, uniquely mapped reads: 57734214, read length: 75bp, type: single-end
sample: iAEC2_DE.ChIP-Seq.H3K27ac.replicate.1.fastq.gz, total number of reads: 43461979, uniquely mapped reads: 40358794, read length: 75bp, type: single-end
sample: iAEC2_DE.ChIP-Seq.H3K27ac.replicate.2.fastq.gz, total number of reads: 51957570, uniquely mapped reads: 49099904, read length: 75bp, type: single-end
sample: iAEC2_DE.ChIP-Seq.Input.replicate.1.fastq.gz, total number of reads: 22962878, uniquely mapped reads: 22042067, read length: 75bp, type: single-end
sample: Cyt49_PP2_shSCRAM.ChIP-Seq.H3K27ac.replicate.1.fastq.gz, total number of reads: 34994822, uniquely mapped reads: 24695846, read length: 75bp, type: single-end
sample: Cyt49_PP2_shPDX1.ChIP-Seq.H3K27ac.replicate.1.fastq.gz, total number of reads: 36796405, uniquely mapped reads: 34018276, read length: 75bp, type: single-end
sample: Cyt49_GT.ChIP-Seq.GATA4.replicate.1.fastq.gz, total number of reads: 26266106, uniquely mapped reads: 22362910, read length: 50bp, type: single-end
sample: Cyt49_GT.ChIP-Seq.GATA4.replicate.2.fastq.gz, total number of reads: 51876343, uniquely mapped reads: 44913914, read length: 75bp, type: single-end
sample: Cyt49_GT.ChIP-Seq.GATA6.replicate.1.fastq.gz, total number of reads: 23211624, uniquely mapped reads: 19727998, read length: 50bp, type: single-end
sample: Cyt49_GT.ChIP-Seq.GATA6.replicate.2.fastq.gz, total number of reads: 31990422, uniquely mapped reads: 27571679, read length: 75bp, type: single-end
sample: H1_WT_PP2_scRNA.fastq.gz, total number of reads: 434652493, uniquely mapped reads: 409007996, read length: 100bp, paired-end
sample: H1_Motif_Optimized_PP2_scRNA.fastq.gz, total number of reads: 432069318, uniquely mapped reads: 409169644, read

	length: 100bp, paired-end
Antibodies	goat anti-FOXA1 Abcam Cat# ab5089, RRID:AB_304744<br>goat anti-FOXA2 Santa Cruz Biotechnology Cat# sc-6554, RRID:AB_2262810<br>mouse anti-HNF4A Novus Cat # PP-H1415, RRID:AB_1964276<br>rabbit anti-H3K27ac Active Motif Cat# 39133, RRID:AB_2561016<br>rabbit anti-H3K4me1 Abcam Cat# ab8895, RRID:AB_306847
Peak calling parameters	HOMER version 4.10.4 was used to call peaks using the findPeaks command with default parameters. The command "-style factor" was used for TFs and the command "- style histone" was used for histone modifications. Stage- and condition-matched input DNA controls were used as background when calling peaks.
Data quality	All peaks were called using the default parameters in the findPeaks program within HOMER suite of bioinformatics tools. The default enrichment and FDR for called peaks is 4-fold and 0.001, respectively.
Software	Burrows-Wheeler Aligner (BWA) version 0.7.13 was used to map ChIP-seq reads to the hg19 reference genome. The findPeaks, makeTagDirectory, getDifferentialPeaks, makeUCSCfile programs from the HOMER suite (v4.8) of bioinformatics tools were used for peak calling, making tag directories, differential peak analysis, and generating bigWig files for visualizing ChIP-seq data.

Flow Cytometry

Plots

Confirm that:

☒ The axis labels state the marker and fluorochrome used (e.g. CD4-FITC).

☒ The axis scales are clearly visible. Include numbers along axes only for bottom left plot of group (a 'group' is an analysis of identical markers).

☒ All plots are contour plots with outliers or pseudocolor plots.

☒ A numerical value for number of cells or percentage (with statistics) is provided.

Methodology

Sample preparation	Cell aggregates derived from hESCs were allowed to settle in microcentrifuge tubes and washed with PBS. Cell aggregates were incubated with Accutase® at room temperature until a single-cell suspension was obtained. Cells were washed with 1 mL ice-cold flow buffer comprised of 0.2% BSA in PBS and centrifuged at 200 g for 5 min. BD Cytofix/Cytoperm™ Plus Fixation/Permeabilization Solution Kit was used to fix and stain cells for flow cytometry according to the manufacturer's instructions. Briefly, cell pellets were re-suspended in ice-cold BD fixation/permeabilization solution (300 µL per microcentrifuge tube). Cells were incubated for 20 min at 4 °C. Cells were washed twice with 1 mL ice-cold 1X BD Perm/Wash™ Buffer and centrifuged at 10 °C and 200 x g for 5 min. Cells were re-suspended in 50 µL ice-cold 1X BD Perm/Wash™ Buffer containing diluted antibodies, for each staining performed. Cells were incubated at 4 °C in the dark for 1 hr. Cells were washed with 1.25 mL ice-cold 1X BD Wash Buffer and centrifuged at 200 g for 5 min. Cell pellets were re-suspended in 300 µL ice-cold flow buffer.
Instrument	FACSCanto™ (BD Biosciences)
Software	FlowJo v10 software https://www.flowjo.com/solutions/flowjo, RRID: SCR_008520
Cell population abundance	Data for 10,000 events in the post-sorted fraction were recorded for each sample. Purity was determined as falling within gated regions determined to exclude negative control (isotype-stained) samples.
Gating strategy	Negative isotype controls were used for all flow cytometry samples. Each sample was split evenly and stained with isotype controls for the same fluorophore(s) used for the target protein staining. Gating strategy excluded regions detected in isotype controls to reduce background as much as possible for target-protein-stained samples.

☒ Tick this box to confirm that a figure exemplifying the gating strategy is provided in the Supplementary Information.
